# Sparse EEG/MEG source estimation via a group lasso

**DOI:** 10.1371/journal.pone.0176835

**Published:** 2017-06-12

**Authors:** Michael Lim, Justin M. Ales, Benoit R. Cottereau, Trevor Hastie, Anthony M. Norcia

**Affiliations:** 1 Department of Statistics, Stanford University, Stanford, CA, United States of America; 2 School of Psychology & Neuroscience, University of St Andrews, Scotland, United Kingdom; 3 Universite de Toulouse, Centre de Recherche Cerveau et Cognition, Toulouse, France; 4 Centre National de la Recherche Scientific, Toulouse Cedex, France; 5 Department of Statistics, Stanford University, Stanford, CA, United States of America; 6 Department of Psychology, Stanford University, Stanford, CA, United States of America; University of Minnesota, UNITED STATES

## Abstract

Non-invasive recordings of human brain activity through electroencephalography (EEG) or magnetoencelphalography (MEG) are of value for both basic science and clinical applications in sensory, cognitive, and affective neuroscience. Here we introduce a new approach to estimating the intra-cranial sources of EEG/MEG activity measured from extra-cranial sensors. The approach is based on the group lasso, a sparse-prior inverse that has been adapted to take advantage of functionally-defined regions of interest for the definition of physiologically meaningful groups within a functionally-based common space. Detailed simulations using realistic source-geometries and data from a human Visual Evoked Potential experiment demonstrate that the group-lasso method has improved performance over traditional *ℓ*_2_ minimum-norm methods. In addition, we show that pooling source estimates across subjects over functionally defined regions of interest results in improvements in the accuracy of source estimates for both the group-lasso and minimum-norm approaches.

## Introduction

Non-invasive recordings of human brain activity through electroencephalography (EEG) or magnetoencelphalography (MEG) provide high-temporal resolution measures of neural activity. When combined with inverse modeling techniques, they also provide information about the underlying distribution of neural activity. The first approach to electromagnetic source localization involved fitting of a single equivalent current dipole to scalp EEG measurements [1, 2]. Starting in the 1990’s, distributed inverse solutions based on the minimum *ℓ*_2_ norm approach (also known as ridge regression) began to appear [3–6]. These methods model the underlying source distribution as a large set of elementary currents, either distributed throughout the intra-cranial volume, or constrained to gray matter. Because the distributed inverse problem is heavily under-determined, there are infinitely many solutions that will recreate the observed signal perfectly. Regularized methods are able to circumvent this problem by penalizing the estimated coefficients, so that one obtains not just a unique source solution, but one that is also more sensible. The *ℓ*_2_ penalty is based on source power: many weakly activated sources are preferred over fewer but stronger sources [7]. Because of this, *ℓ*_2_ minimum-norm solutions are blurry and contain inverted sign “ghost sources” that are not present in the actual source distribution, even under no noise conditions [8, 9].

A second major approach to the distributed source modeling problem has been a range of empirical Bayes methods [10–13]; (see [14] and [15] for reviews). In the context of source-estimation, a key concept that links norm-based approaches and Bayesian approaches is the assumed prior [14]. These are typically Gaussian priors, and their covariances are used to impose spatial and temporal smoothness, along with a level of agreement in the case of multiple subjects. The prior covariances can be specified up to a number of free hyper-parameters. These can also be controlled via prior distributions, but the more pragmatic empirical Bayes techniques estimate them using the data at hand.

While it is true that there is often a direct correspondence between a regularized fit and a Bayes posterior mode (*e.g.* ridge regression and Gaussian prior, lasso regression and Laplacian prior), the regularization approach has several practical advantages over the Bayesian approach, since it allows for more transparency and flexibility. As just noted above, the minimum *ℓ*_2_-norm approach assumes a Gaussian prior. Following the early work on distributed source imaging with the *ℓ*_2_-norm, source localization methods based on penalty functions with *L*_*p*_ norms where *p* < 2 were introduced [16–20]. The *ℓ*_1_ (lasso) penalty [21] assumes a Laplacian or double exponential prior consistent with an assumption that there are only a small number of highly active sources. Methods that use *ℓ*_1_ penalty result in “sparse” estimates of the sources where only a small number of them are nonzero. This has the advantage of being able to produce estimates that are highly localized. However, these approaches can have unstable location estimates, and this has limited their wide-spread application. The susceptibility to noise and independent estimation at each time-point causes the highly focal recovered sources to shift unpredictably from locus to locus over time [19, 22]. Spatial smoothing can alleviate the instability of *ℓ*_1_-penalized methods, but at the expense of the focality of the source estimate. Alternatively, temporal constraints can be imposed to promote smoothness without sacrificing focality [12, 14, 23–26]. Finally, a more recent development within the *L*_*p*_ norm approach is to use an elastic-net type of penalty [27–30] These penalties employ a combination of *ℓ*_1_ and *ℓ*_2_ penalties to reap the benefits that each has to offer. They retain the sparsity of the recovered sources that a pure *ℓ*_1_ penalty provides, while the *ℓ*_2_ penalty serves as a smoother that takes care of the instabilities in the *ℓ*_1_ solution.

The norm-based and empirical Bayes approaches have largely been applied in the context of single-subject source recovery with pooling of information across subjects being accomplished as a post-processing step. One reason for this is that norm-based methods are inherently unable to pool information across multiple subjects. For example, the *ℓ*_2_ minimum-norm approach on *S* subjects decouples into *S* individual minimum-norm problems, each of which can be solved independently of the others. Here, one could average the recovered sources across subjects to get a final estimate [31–33]. Another recently proposed method uses the topographic maps within visual areas to setup cross-subject correspondence [34].

Hierarchical Bayes models can account for structure at different levels (within a subject and between subjects), and can be quite general. However, one pays a price for this complexity. The specification of the models is complex, and the algorithms for fitting them do not scale well as the number of parameters grow. We prefer regularization over the Bayesian approach, since it allows for more transparency and flexibility.

Instabilities in traditional sparse solutions at the individual subject level also pose a difficulty when one wishes to perform multi-subject analyses in a common anatomical framework such as a template brain: individual, highly sparse activations tend to not overlap in the common space, leading to low levels of statistical significance when statistical parametric mapping approaches are used. A previous solution to this problem uses a hierarchical Bayes technique that fits a Gaussian process with a choice of kernel that imposes group structure [15, 35]. This framework also utilizes a common anatomical space for inversion in which a template cortical surface is fit to the brains of each individual subject [36]. Aligning individual brains to a common template removes some, but not all of the individual variability associated with the location of functional brain areas with respect to gross features of the cortex surface or volume.

An alternative approach to the common space that has higher specificity and functional interpretability is to use functional Magnetic Resonance Imaging (fMRI) to map cortical areas that exist independently of the activation under test [31, 32, 37, 38]. In particular, the visual system contains a series of topographically organized maps of the contralateral visual field in each hemisphere [39]. These topographically organized areas are present in each individual and have different functional specializations [40–42]. Previous work has exploited the topographic organization within individual subjects to improve time course estimates [43, 44]. Those methods work by stimulating multiple locations on the topographic maps and use the known organization to constrain the optimization. However, the requirement for specific, topographic organization is limiting. Nonetheless constraints based on additional functional brain areas or Regions of Interest (ROIs) that can be defined on the basis of fMRI localizer tasks in which the areas are defined in terms of their functional specialization, rather than on topographic criteria [45–47] are likely to be useful. These two factors, topographic organization and functional specialization together form an independent, rationale basis for comprising features, or importantly for what we propose, groups of features.

In the approach we propose here, we use a combination of rank-reduction and group-lasso penalization to select activations at the ROI level that ensures a form of agreement among subjects as to which particular features should be chosen for the solution. It is thus a generalization of the elastic-net approach: groups of features are comprised from sources at vertices within a given ROI. The method we propose here also enforces group-level consistency of sources across subjects via a sparse, group-level penalty on the active ROIs. There are thus two senses in which we use the term “group” one is the grouping of features within the fMRI ROIs and the other is at the level of group analysis of data from multiple subjects. The result is an improvement in source recovery beyond what can be obtained by simple averaging of individual source estimates. Because we define ROIs on the basis of functional fMRI mapping of visual areas, the ROIs provide a functionally meaningful way of defining sources as they are based on either topographic or functional criteria that are independent of the source estimation. Moreover our method does not warp individual subject’s brains to a template brain, rather the focus is on individually mapped ROIs as a more realistic and geometrically accurate source space. This kind of model crafting is more difficult with the Bayesian approach.

Using realistic simulations, we show that group lasso inversion, operating on functional ROIs, improves source recovery above and beyond what can be accomplished with the classical minimum norm for single subjects. We also show that both the minimum norm and group-lasso estimates based on functional ROI constraints improve with increasing numbers of subjects. This improvement is distinct from the effect of group-variable selection across subjects and is more pronounced for the group lasso than it is for the minimum norm. We begin with a brief conceptual overview of the method, followed by a detailed description of the algorithm. We then evaluate our method using realistic simulations and make comparisons with the classical minimum-norm solution. Finally, we compare our method to the minimum norm on a human Visual Evoked Potential data set.

## Materials and methods

**Fig 1:**
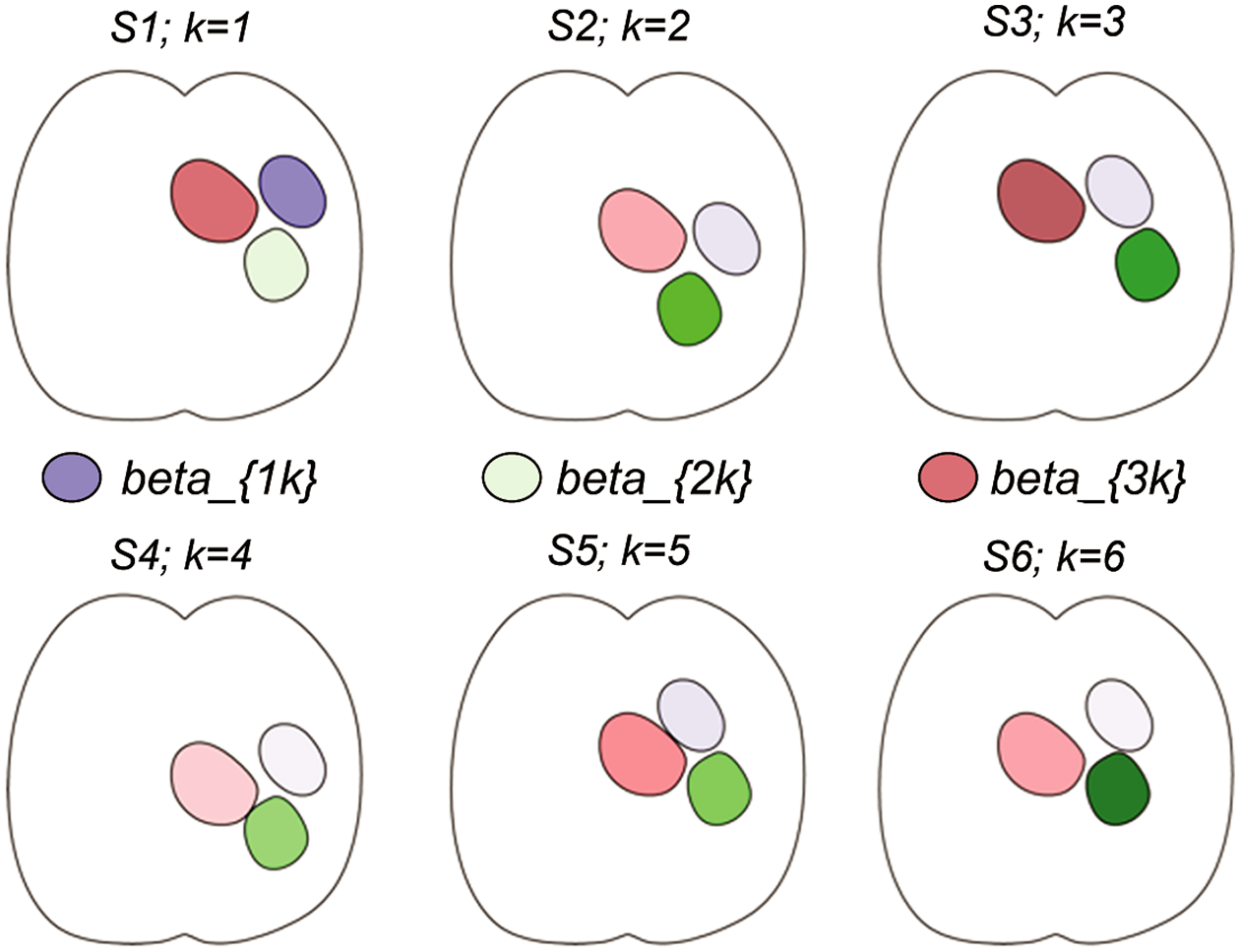
Schematic of the group-lasso settling disputes. The true areas are shaded pink and green. The blue region is stronger than green in subject 1, but pink and green still get chosen over the blue because of their aggregate strength across the other 5 subjects; in effect, a majority vote. In the group-lasso solution, the blue ROI activation would be set to zero.

The logic of the group-lasso approach to source inversion can be illustrated by the simplified schematic example shown in Fig 1. A detailed description of our methods for ROI definition and the algorithm follow. In the schematic example, we illustrate the case of three ROIs, whose source activity is labelled as *β*_1*k*_, *β*_2*k*_, *β*_3*k*_. The three ROIs are of different sizes and have different shapes and locations across six example brains (individual subjects are indexed by k). The reconstruction problem is to localize the activity to the correct regions. In reality, only the 2nd (green) and 3rd (pink) ROIs are active. The strength of the shading in the diagram indicates the strength of the recovered signal (beta) in each subject. Due to different positioning and aliasing in their separate forward matrices, in some subjects some of this activation is attributed erroneously to the inactive, 1st ROI (purple). In particular, in subject 1 the individual subject source reconstruction recovered activity in first ROI that is stronger than in the 2nd, actually active ROI, which in this simulation is an error. The group lasso ties the corresponding ROIs across subjects together. It decides collectively, for example, that the 2nd ROI is active, in which case it will be active in *all* subjects (albeit at different strengths in each). In this case, since the recovered activity in the 1st ROI is mostly weak across the group, the 1st ROI would be set to zero by the group penalty, and the model would correctly recover the actually active 2nd and 3rd regions.

### The group lasso inversion algorithm

We first set forth notation that will be used throughout this paper. We define 18 ROIs per subject as noted below. Let *p*_*i*_, *i* = 1, …, 18 denote the number of vertices in the *i*-th ROI, and let **F**_*i*_, *i* = 1, …, 18 denote the forward matrix for the *i*-th ROI. We use **Y** to represent the *N* × *T* matrix consisting of *N* sensor observations at *T* time points, and ***β***_*i*_ is the *p*_*i*_ × *T* matrix of neural activity in the *i*-th ROI that we wish to recover. The overall forward matrix is denoted by

$$\begin{array}{c} F=[F_{1},F_{2},\ldots ,F_{18}], \end{array}$$ (1)

and

$$\begin{array}{c} \beta =[\begin{array}{c} \beta_{1}\\ \beta_{2}\\ \vdots \\ \beta_{18} \end{array}] \end{array}$$ (2)

is the overall matrix of neural activity. When referring to multiple subjects, we use superscripts to index the subject, so that $F_{i}^{k}$ is the $N\times p_{i}^{k}$ forward matrix for subject *k*’s *i*-th ROI, and similarly for the overall forwards **F**^*k*^. Note that $F_{i}^{k}$ and $F_{i}^{l}$ can have different numbers of columns; there is in general no correspondence between the individual elements of $\beta_{i}^{k}$ and $\beta_{i}^{l}$.

The forward model that relates the neural activity to the sensor observations can now be expressed as

$$\begin{array}{ccc} Y & = & F\beta +\epsilon \end{array}$$ (3)

$$\begin{array}{ccc} & = & \sum_{i=1}^{18}F_{i}\beta_{i}+\epsilon , \end{array}$$ (4)

where **ϵ** is a noise term, typically assumed to be distributed as *N*(0, *σ*^2^ **I**).

Recovering the neural activity is an example of an ill-posed inverse problem (commonly referred to as the “*p* > *n*” problem in statistics) where there are more parameters or variables than observations. A popular approach in supervised learning problems of this type is to use regularization, such as adding a squared *ℓ*_2_ penalty of the form ${∥\beta ∥}_{2}^{2}$ or a *ℓ*_1_ penalty of the form ‖***β***‖_1_ to the coefficients. This “lasso” penalty has been the focus of much research since its introduction [21]. One of the reasons for the lasso’s popularity is that it does variable selection: it sets some coefficients exactly to zero. Lasso used directly in EEG/MEG source recovery leads to spotty solutions lacking spatial coherence, exacerbated by the high correlations between columns of the forward matrix. Here we use instead an analog of the lasso, called the group-lasso [48], that sets groups of variables to zero. In our application, each ROI defines a group of sources located at the vertices of each ROI. This approach exploits the prior information that groups (ROIs) will tend to be entirely off or mostly on. A plain lasso would respond to noise and set perhaps many spurious sources on, even in regions where there is no activation (minimum norm has every voxel on, albeit some weakly). By exploiting this prior information, the group lasso can spend its degrees of freedom for fitting more wisely.

We first describe the generic group lasso for vector-valued coefficients. Suppose there are *p* groups of variables (possibly of different sizes), and let the feature matrix for group *i* be noted by **X**_*i*_ (for us **X**_*i*_ could be **F**_*i*_ or transformations thereof). Let **Y** denote the vector of observations. The group-lasso obtains the estimates ${\hat{\beta }}_{j}$ as the solution to

$$\begin{array}{c} {\text{argmin}}_{\mu ,\beta }\frac{1}{2}{∥Y-\mu \cdot 1-\sum_{i=1}^{p}X_{i}\beta_{i}∥}_{2}^{2}+\lambda \sum_{i=1}^{p}\gamma_{i}{∥\beta_{i}∥}_{2}, \end{array}$$ (5)

where *μ* is an intercept term. The first term controls the fit of the model to the data, while the second term controls the complexity of the fit: in this case both the number of active ROIs and the range of activity in those selected. The second term is a penalty on the sources. Here ‖***β***_*i*_‖_2_ is an *ℓ*_2_ norm, the square-root of the sum-of squares of the components in ***β***_*i*_. The penalty weights *γ*_*i*_ allow us to modify the relative amount of penalization for the *i*th ROI, and *λ* is an overall penalty-strength parameter. The nature of this penalty is that some of the ***β***_*i*_ will be estimated to be exactly zero (i.e. an entire ROI), and some not; for those ROIs that are non-zero, all their vertex-level activities are typically non-zero. If by contrast we used ${∥\beta_{i}∥}_{2}^{2}$ in each of the penalty terms, rather than their square roots, the solution would be entirely nonzero, and equivalent to the class of minimum-norm estimates. If each group were size one—e.g. a single source—then ‖***β***_*i*_‖_2_ = |*β*_*i*_|, and the group lasso reduces to the ordinary lasso. Solving Eq (5) is a convex optimization problem; in principal any solver can be used, but the structure of the problem lends itself to certain efficient implementations that we have used.

The parameter *λ* controls the amount of regularization, with larger values implying more regularization (and hence more groups of coefficients being set to zero). The *γ*_*i*_’s allow each group to be penalized to different extents; we take *γ*_*i*_ = ‖**X**_*i*_‖_*F*_, the Frobenius norm of the feature matrix for group *i* (see S1 Algorithm Details). To solve Eq (5), we start with *λ* large enough so that all estimates are zero. Decreasing *λ* along a grid of values results in a path of solutions from which an optimal *λ* can be chosen using cross validation or some other model selection procedure; we use generalized cross validation (GCV) [49].

It is often useful to mix the group-lasso penalty with the fully quadratic (minimum norm) penalty when the set of features are highly correlated. Adding this quadratic penalty to Eq (5) results in the group analog of the elastic-net generalization of the lasso:

$$\begin{array}{c} {\text{argmin}}_{\mu ,\beta }\frac{1}{2}{∥Y-\mu \cdot 1-\sum_{i=1}^{p}X_{i}\beta_{i}∥}_{2}^{2}+\lambda \sum_{i=1}^{p}\gamma_{i}{∥\beta_{i}∥}_{2}+\alpha {∥\beta ∥}_{2}^{2}. \end{array}$$ (6)

The squared *ℓ*_2_ penalty in Eq (6) applies to the entire coefficient vector ***β***. This allows us to reduce the variance in the estimates ${\hat{\beta }}_{i}$, and having *α* > 0 is helpful in our experiments. Notice that if *λ* = 0, this is equivalent to a minimum-norm objective. Now that we have two parameters *λ* and *α*, in principal a two-dimensional grid search would have to be done to select optimal values for them. Since this can be computationally intensive, we keep *α* fixed and do the grid search only on *λ*. We discuss how we select *α* in S1 Model Selection Details.

### Extending the group-lasso to matrix-valued coefficients

Our description of the group-lasso above treats the coefficients ***β*** as a vector. Since the neural activity at a single time point is a vector, and we wish to recover the activity over several time points, we need to be able to handle the case where the coefficients are matrices. This can be done via a straightforward extension of Eq (6). As before let **X**_*i*_ denote the feature matrix for group *i* and let **Y** be the *N* × *T* *matrix* of observations.

$$\begin{array}{c} {\text{argmin}}_{\mu ,\beta }\frac{1}{2}{∥Y-1\mu^{t}-\sum_{i=1}^{p}X_{i}\beta_{i}∥}_{F}^{2}+\lambda \sum_{i=1}^{p}\gamma_{i}{∥\beta_{i}∥}_{F}+\alpha {∥\beta ∥}_{F}^{2}. \end{array}$$ (7)

We now have *T* intercepts in ***μ*** (one for each column of **Y**), and the coefficients ***β***_*i*_ are *T*-column matrices, and we use the Frobenius norm ‖ ⋅ ‖_*F*_ instead of the *ℓ*_2_ norm. The solutions have the same property as before in that if ${\hat{\beta }}_{i}$ is nonzero, then *all* its components are usually nonzero. Details for obtaining solutions to Eq (7) are provided in S1 Algorithm Details.

### Improved selection using multiple subjects

As noted in the Introduction, the “all zero or all nonzero” property of the group-lasso allows us to pool information across multiple subjects, leading to improved accuracy in identifying the active ROIs. One way to make use of the data from multiple subjects is to build a large forward matrix by stacking the individual matrices from each subject, and similarly for the observations. Stacking multiple subjects into one forward matrix has been shown to improve estimates [15, 34]. But to accomplish the stacking properly requires the ability to create a strong correspondence between sources across subjects. This is possible in visual areas that have a strong topographic organization which enables a 1-1 correspondence between source locations across subjects. The method proposed here goes further and is usable even in regions without such a strong correspondence, for example in ROI’s of different sizes. We also want to impose both spatial (across vertices) and temporal smoothness in the recovered activity. The dimension reduction that results from smoothing also leads to computational speedups.

Because column *c* of a subject’s forward matrix measures the contribution of vertex *c* to each of the *N* sensors, we expect neighboring vertices to have roughly the same contribution, that is, the contributions should vary smoothly as we traverse the vertices in a ROI. We thus expect the forward matrices **F**_*i*_ Eq (1) to be low rank where most of the variation can be captured by the top few principal components. We use 5 components per ROI because the orientation of a ROI can be parametrized with 3 spatial coordinates along with 2 rotation angles, and this seems to work well in our experiments. This method of spatial smoothing respects the borders of the functional areas: smoothing does not occur across areas that may differ in their functional specificity, as might happen with a purely spatial smoothing such as that used in LORETA [5].

Recall that $F_{i}^{k}$ denotes the $N\times p_{i}^{k}$ forward matrix of subject *k* that corresponds to ROI *i*. Let $P_{i}^{k}$ denote the $p_{i}^{k}\times 5$ matrix consisting of the first 5 right singular vectors of the centered $F_{i}^{k}$ (column means removed). The columns of $P_{i}^{k}$ can be viewed as a smooth basis across the space of vertices in ROI *i*, inheriting the smoothness represented in $F_{i}^{k}$). Hence we can impose a similar spatial smoothness on the recovered activity by constraining $\beta_{i}^{k}$ to be a linear expansion in this basis:

$$\begin{array}{c} \beta_{i}^{k}=P_{i}^{k}{\beta^{\prime }}_{i}^{k}, \end{array}$$ (8)

where ${\beta^{\prime }}_{i}^{k}$ is now the 5-vector of coefficients representing $\beta_{i}^{k}$. The observed signal contribution from ROI *i* in subject *k* can then be written as

$$\begin{array}{ccc} F_{i}^{k}\beta_{i}^{k} & = & F_{i}^{k}P_{i}^{k}{\beta^{\prime }}_{i}^{k}\\ & = & X_{i}^{k}{\beta^{\prime }}_{i}^{k}, \end{array}$$ (9)

where

$$\begin{array}{c} X_{i}^{k}=F_{i}^{k}P_{i}^{k} \end{array}$$ (10)

is the *N* × 5 matrix consisting of the first 5 principal components of $F_{i}^{k}$. We call $X_{i}^{k}$ the *filtered forward matrix* for ROI *i* in subject *k* (see [15]). The overall filtered forward matrix for *S* subjects can then be constructed by

$$\begin{array}{c} X=[\begin{array}{ccccccccc} X_{1}^{1} & & & & \cdots & X_{18}^{1} & & & \\ & X_{1}^{2} & & & \cdots & & X_{18}^{2} & & \\ & & \ddots & & \cdots & & & \ddots & \\ & & & X_{1}^{S} & \cdots & & & & X_{18}^{S} \end{array}]. \end{array}$$ (11)

We can write the (*N* ⋅ *S*) × (5 ⋅ *S* ⋅ 18) matrix **X** in more compact form (recall that subscripts index ROIs and superscripts index subjects) by

$$\begin{array}{c} X=[X_{1},X_{2},\ldots ,X_{18}], \end{array}$$ (12)

where $X_{i}=[\begin{array}{cccc} X_{i}^{1} & & & \\ & X_{i}^{2} & & \\ & & \ddots & \\ & & & X_{i}^{S} \end{array}].$

Combining observations from various subjects is more straightforward, and can be done by simply stacking the observations:

$$\begin{array}{c} Y=[\begin{array}{c} Y^{1}\\ Y^{2}\\ \vdots \\ Y^{S} \end{array}]. \end{array}$$ (13)

The group-lasso objective for the filtered forward matrices **X**_*i*_ and the spatially smoothed activity coefficients ***β***′ now be written as

$$\begin{array}{c} {\text{argmin}}_{\mu ,\beta^{\prime }}\frac{1}{2}{∥Y-1\mu^{t}-\sum_{i=1}^{18}X_{i}\beta_{i}^{\prime }∥}_{F}^{2}+\lambda \sum_{i=1}^{18}\gamma_{i}{∥\beta_{i}^{\prime }∥}_{F}+\alpha {∥\beta^{\prime }∥}_{F}^{2}. \end{array}$$ (14)

This is because of Eqs (8) and (9), and the fact that the $P_{i}^{k}$ are orthogonal, this criterion is equivalent to a similar one using the higher dimensional **F**_*i*_ and ***β***_*i*_. Here each $\beta_{i}^{\prime }$ has dimension (*S* ⋅ 5) × *T*. Because a group now consists of a single ROI across multiple subjects, there is a collaborative effect in that as long as a ROI has a strong signal in enough subjects, we will estimate that ROI to be nonzero even in those subjects where that ROI is not quite lighting up. We expect this pooling effect to be stronger as the number of subjects increases, and we show in the Results that this is indeed the case.

It is more difficult to pool information across subjects using the minimum norm or elastic-net approaches. Any such pooling is typically done manually as a post-processing step, such as averaging the estimated sources over the multiple subjects.

### Imposing temporal smoothness

In the spirit of the previous section, it is reasonable to assume that the neural activity also varies smoothly over time, and we can impose temporal smoothness in the estimated source by finding a suitable basis for the time component. The right singular vectors of **Y** are a natural basis for the temporal component:

$$\begin{array}{c} Y_{N\times T}=U_{N\times N}D_{N\times T}V_{T\times T}^{t}. \end{array}$$ (15)

The singular value decomposition is also used in [12] to obtain the principal directions along the time axis, but there they use 5 singular vectors. We fix the dimension *d* of this basis by taking as many singular vectors as we need to explain 99% of the variance of *Y*. In particular, this is given by

$$\begin{array}{c} d={\text{argmin}}_{k}\left\{k:\frac{\sum_{i=1}^{k}d_{ii}^{2}}{\sum_{i=1}^{N}d_{ii}^{2}}\geq 0.99\right\}. \end{array}$$ (16)

In our experiments, this number is typically 2. Let **V**_*d*_ be the matrix consisting of the first *d* columns of **V**. We restrict each $\beta_{i}^{\prime }$ to the space spanned by **V**_*d*_ by setting

$$\begin{array}{c} \beta_{i}^{\prime }={\tilde{\beta }}_{i}V_{d}^{t}, \end{array}$$ (17)

where the ${\tilde{\beta }}_{i}$ each have dimension (*S* ⋅ 5) × *d*.

Thus, after spatial and temporal filtering, we have, for *S* subjects, the *NS* × *d* matrix $\tilde{Y}$ of filtered observations, the (*N* ⋅ *S*) × (*S* ⋅ 18 ⋅ 5) filtered forward matrix **X**, and (*S* ⋅ 18 ⋅ 5) × *d* matrix $\tilde{\beta }$ of filtered activity that we need to estimate. This is achieved by solving Eq (19).

Applying restriction Eqs (17) to (14) gives

$$\begin{array}{c} {\text{argmin}}_{\mu ,\tilde{\beta }}\frac{1}{2}{∥Y-1\mu^{t}-\sum_{i=1}^{p}X_{i}{\tilde{\beta }}_{i}V_{d}^{t}∥}_{F}^{2}+\lambda \sum_{i=1}^{p}\gamma_{i}{∥{\tilde{\beta }}_{i}V_{d}^{t}∥}_{F}+\alpha {∥\tilde{\beta }V_{d}^{t}∥}_{F}^{2}. \end{array}$$ (18)

Because the columns of **V**_*d*_ are orthonormal, this is equivalent to minimizing

$$\begin{array}{c} {\text{argmin}}_{\tilde{\mu },\tilde{\beta }}\frac{1}{2}{∥\tilde{Y}-1{\tilde{\mu }}^{t}-\sum_{i=1}^{p}X_{i}{\tilde{\beta }}_{i}∥}_{F}^{2}+\lambda \sum_{i=1}^{p}\gamma_{i}{∥{\tilde{\beta }}_{i}∥}_{F}+\alpha {∥\tilde{\beta }∥}_{F}^{2}, \end{array}$$ (19)

where $\tilde{Y}=YV_{d}$ is the *N* × *d* matrix of temporally filtered observations.

### Recovering the activity in the original space

Once we obtain an estimate of $\hat{\tilde{\beta }}$ from Eq (19), we can transform it back to the original space by reversing the temporal filtering and dimension reduction operations. We illustrate for a single subject. Let

$$\begin{array}{c} P^{k}=[\begin{array}{ccc} P_{1}^{k} & & \\ & \ddots & \\ & & P_{18}^{k} \end{array}] \end{array}$$ (20)

denote the block diagonal matrix consisting of the $P_{i}^{k}$’s in Eq (8). From Eq (8), it is clear that reversing the spatial filtering can be done by left-multiplying our solution $\hat{{\tilde{\beta }}^{k}}$ by **P**. Similarly, Eq (17) shows that right-multiplying by ${V_{d}^{k}}^{t}$ reverses the temporal filtering. To summarize, our smoothed estimate of the source activity in the original space is given by

$$\begin{array}{c} \hat{\beta^{k}}=P^{k}\hat{{\tilde{\beta }}^{k}}{V_{d}^{k}}^{t}. \end{array}$$ (21)

### Model selection

Generalized cross validation (GCV) is one method of model selection that is intuitively simple and widely used. Let **Y** be the *N* × *T*-matrix of observations, and $\hat{Y}$ the fitted values. The GCV error for this fit is given by

$$\begin{array}{c} \frac{\frac{1}{NT}{∥Y-\hat{Y}∥}_{F}^{2}}{{\left(1-\frac{df(\hat{Y})}{NT}\right)}^{2}}, \end{array}$$ (22)

where $df(\hat{Y})$ is the degrees of freedom for $\hat{Y}$. Fitting the group lasso along a grid of *λ* values results in a GCV error curve. We then pick the *λ* that gives the minimum value on this curve. Details on approximating the degrees of freedom for the group-lasso solutions and selecting the *α* parameter in Eq (7) are given in S1 Model Selection Details.

### Defining regions of interest (ROIs) in the visual cortex

As noted above, grouping of features for group-lasso estimation benefits from a rational basis for defining the groups and here we exploit the existence of multiple functional maps in the visual cortex to comprise the basis for group formation. For purposes of the present analysis, we defined the detailed 3D shape of each of 18 visual ROIs in 25 participants (V1-L, V1-R, V2v-L, V2v-R, V2d-L, V2d-R, V3v-L, V3v-R, V3d-L, V3d-R, V4-L, V4-R, V3A-L, V3A-R, LOC-L, LOC-R, MT-L, MT-R). These definitions are based on high-resolution T1 anatomical scans combined with functional MRI scans. Structural and functional MRI scanning was conducted at 3T (Siemens Tim Trio, Erlangen, Germany) using a 12-channel head coil. We acquired a T1-weighted MRI dataset (3-D MP-RAGE sequence, 0.8 × 0.8 × 0.8*mm*^3^ and a 3-D T2-weighted dataset (SE sequence at 1 × 1 × 1*mm*^3^ resolution) for tissue segmentation and registration with the functional scans. For fMRI, we employed a single-shot, gradient-echo EPI sequence (TR/TE = 2000/28 ms, flip angle 80, 126 volumes per run) with a voxel size of 1.7 × 1.7 × 2*mm*^3^ (128 × 128 acquisition matrix, 220 mm FOV, bandwidth 1860 Hz/pixel, echo spacing 0.71 ms). We acquired 30 slices without gaps, positioned in the transverse-to-coronal plane approximately parallel to the corpus callosum and covering the whole cerebrum. Once per session, a 2-D SE T1-weighted volume was acquired with the same slice specifications as the functional series in order to facilitate registration of the fMRI data to the anatomical scan. The research was reviewed and approved by the Institutional Review Board of Stanford University. Informed, written consent was obtained from each participant prior to the imaging study.

The FreeSurfer software package (http://surfer.nmr.mgh.harvard.edu) was used to perform gray and white matter segmentation to define a cortical surface mesh with accurate surface normals. The FreeSurfer package extracts both gray/white and gray/cerebrospinal fluid (CSF) boundaries, but these surfaces can have different surface orientations. In particular, the gray/white boundary has sharp gyri (the curvature changes rapidly) and smooth sulci (slowly changing surface curvature), while the gray/CSF boundary is the inverse, with smooth gyri and sharp sulci. We created a new surface that had a similar curvature for both gyri and sulci, avoiding these curvature discontinuities. The new surface generated by interpolating a position that was midway between the gray/white surface and the gray/CSF surface using the FreeSurfer function mris_expand. The tessellation of cortex used for creating sources had 20484 vertices on a decimated cortical surface mesh (decimated from 290,000 vertices in the original T1 anatomical image. The forward matrix in Eq (1) only takes into account sources within the visual ROIs. Each *F*_*i*_ has 128 rows and *n*_*i*_ columns where *n*_*i*_ is the number of sources within the *i*th ROI. The ROI size varied by visual area, with V1 being the largest ROI by area and thus the largest number of columns was devoted to it in the forward matrix. For a given ROI, the size was specific for each individual cortex and was based on their individual mapping results.

The highest accuracy for source-imaging is obtained when there is an accurate model that connects activity at each location on the surface of cortex with how it will be measured at the scalp. To generate realistic scalp topographies, we made separate forward models for each participant in the study using the Boundary Element Method (BEM) with conductivity models that were derived from the T1 and T2 weighted MRI scans of each observer. The FSL toolbox (http://www.fmrib.ox.ac.uk/fsl/) was also used to segment contiguous volume regions for the scalp, outer skull, and inner skull and to convert these MRI volumes into inner skull, outer skull, and scalp surfaces [50].

The general procedures for the scans used to define the visual areas (head stabilization, visual display system, etc) are standard and have been described in detail elsewhere [51]. Retinotopic field mapping defined ROIs for visual cortical areas V1, V2v, V2d, V3v, V3d, V3A, and V4 in each hemisphere [52, 53]. ROIs corresponding to hMT+ were identified using low contrast motion stimuli similar to those described in [54]. In this study, the fMRI data was used purely to define ROIs for the EEG analysis.

### Simulation setup

**Fig 2:**
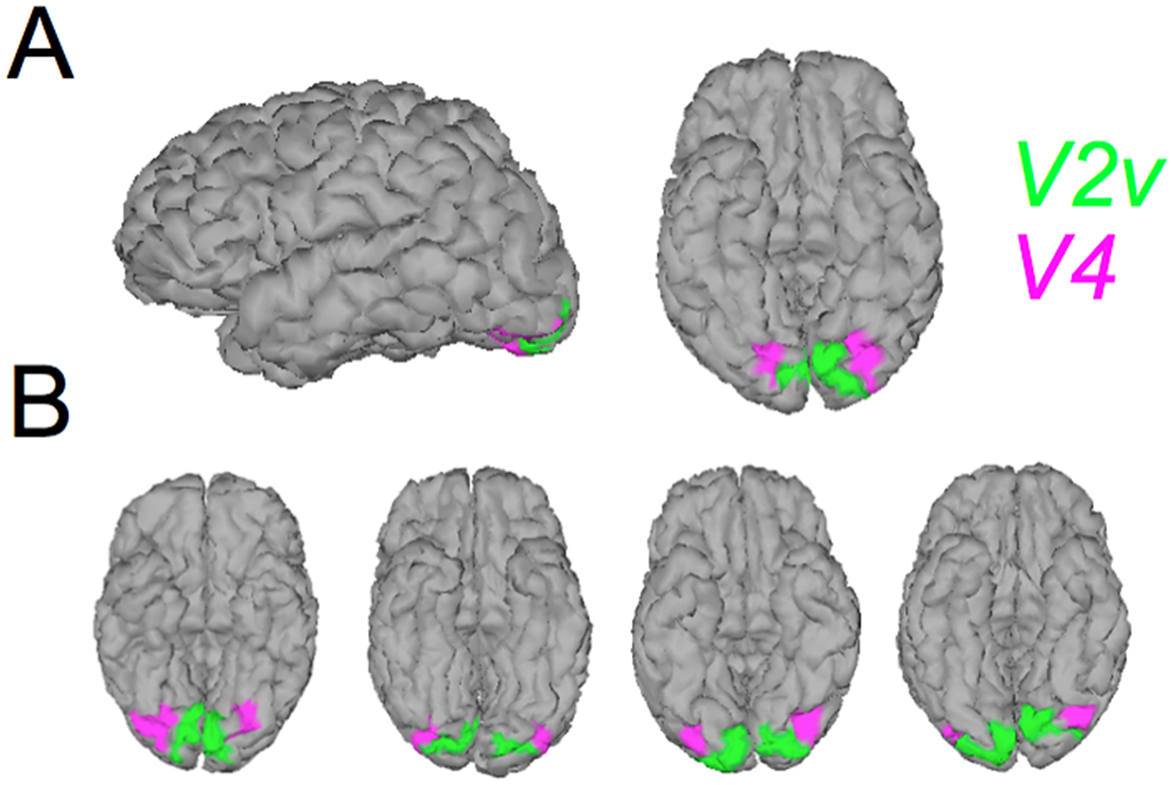
Localization of the visual ROIs used for the simulations. V2v is in green and V4 is in purple. A) Shows left and ventral views of one typical subject. B) Shows the ventral views of 4 other subjects.

Our simulations were generated using the protocol described in a previous paper from our group [55]. We took two retinotopic ROIs of the ventral cortex: V2v and V4. The locations of these ROIs are shown on a representative cortical surface in Fig 2. Within each of these ROIs, we randomly defined contiguous clusters whose surfaces (in *mm*^2^) were equal to 30% of the ROI surfaces. The activations in each cluster were uniform and their amplitudes were randomly chosen between 1 and 10. We then passed the activity through the forward model to obtain the observed time courses **Y**. We added gaussian white noise to **Y** to obtain a signal to noise ratio of 0.32 (defined as Var(**Y**)/Var(noise).) In all cases, we take *N* = 128 observations/sensors and *T* = 91 time points. ROIs V2v and V4 are separated by the V3v ROI. These three ROIs exhibit considerable cross-talk between their forward vectors and the Euclidian distance between the V2v and V4 ROIs is about a centimeter on average (see the examples in Fig 2). In addition, these ROIs lie on the ventral cortical surface and are therefore quite distant from the electrodes. Our simulations therefore constitute very challenging activations to reconstruct from the measurements at the electrode level.

### Source estimation quality metrics

The lasso and conventional minimum norm methods are evaluated on 3 measures (see [55] for more details):

1. **Area under the ROC curve (AUC)**
  The receiver-operating curve (ROC) is an estimator of the detection accuracy [56, 57]. It evaluates the ability of a reconstruction to select as active, only the sources that were actually activated in the simulation. The AUC thus quantifies how well the estimated currents detect true sources and reject false positives. For a given reconstruction, we can define the threshold-dependent values of the specificity *S*_*p*_ and the sensitivity *S*_*e*_:
  $$\begin{array}{c} S_{e}\left(c\right)=\frac{TP(c)}{TP(c)+FN(c)}, \end{array}$$ (23)
  $$\begin{array}{c} S_{p}\left(c\right)=\frac{TN(c)}{TN(c)+FP(c)} \end{array}$$ (24)
  where TP(*c*), FN(*c*), TN(*c*) and FP(*c*) are the true positives, the false negatives, the true negatives, and the false positives corresponding to the threshold *c*. ROC curves are then obtained by plotting *S*_*e*_(*c*) against 1—*S*_*p*_(*c*), which is a monotonically increasing function. The AUC is an index of the specificity-sensitivity compromise of the corresponding model. An AUC close to 1 means that the model separates the active and nonactive sets of sources well. However, in our simulations, the number of inactive sources is very much larger than the number of active sources. Because only a few percent of the sources are true positives, a trivial solution that estimates zero everywhere would have a high correct reject rate that leads to a biased estimation of the false positive rate. To circumvent this problem, we defined subsets where the number of inactive sources is strictly equal to the number of active ones (see [55]):
  - a set that includes the *n* active sources in a simulation and their *n* closest neighbors. The associated ROC curve allows us to define the *AUC*_*close*_ value that quantifies the focalization ability of the models by estimating their ability to separate between active and nonactive sources in the closest neighborhood of the activity.
  - A set that includes the *n* active sources in a simulation and the *n* sources outside the neighborhood of the activation whose activities are the highest. The associated ROC curve permits to define the *AUC*_*far*_ value that quantifies the ability of an estimator to discriminate between the real activated sources and the local maxima localized far from the simulated set.
  In the end, a global AUC value can be computed as an average of these two scalars:
  $$\begin{array}{c} AUC=1/2(AUC_{close}+AUC_{far}) \end{array}$$ (25)
2. The **mean squared error (MSE)** on the neural activity is given by $\frac{1}{NT}{∥\beta -\hat{\beta }∥}_{F}^{2}$. It reflects the fit to the ground-truth signal.
3. The **relative energy** is given by the ratio between the normalized energies contained in the estimate of the active sources and the global distribution:
  $$\begin{array}{c} \frac{\sum_{i\in A}E_{est}\left(i\right)}{\sum_{i}E_{est}\left(i\right)}, \end{array}$$ (26)
  where A is the set of active vertices in the true neural activity and *E*_*est*_(*i*) is the energy of the estimated signal at vertex *i*. This is a measure of the extent to which the correct sources are identified.

For a single subject, we compute these metrics for each of the *T* time points, then take the average. For multiple subjects, we compute this time average separately for each subject, and then take the average across all subjects.

### Real data collection

Visual Evoked Potentials were recorded from 9 adult observers with normal visual acuity and stereopsis. The participants viewed a display consisting of dynamic random-dot kinematograms that alternated at 1 Hz between coherent and incoherent motion states. The coherent state consisted of rotary coherent motion for 500 msec, alternated with incoherent motion for 500 msec. The direction of coherent motion alternated between being clock-wise vs counter-clockwise so as to reduce the effects of motion adaptation. A full cycle of the stimulus thus lasted two seconds and 5 cycles of stimulation were presented as 10 sec trials (n = 10), with 1 sec of additional presentation at the beginning to allow for start-up transients associated with the onset of the dots from a blank screen. The dots were updated at 30 Hz.

## Results

We first make comparisons between the group-lasso and minimum-norm methods through detailed simulation with realistic source configurations for the visual cortex. We also evaluate the methods on multiple subjects (up to 25), and demonstrate that the effectiveness of the group lasso increases with the number of subjects. The minimum-norm method does not inherently pool information across multiple subjects, but we can average the recovered activity across subjects for each ROI as a post-processing step. This ROI-based averaging improves performance for both the group lasso and the minimum norm. We then describe a comparison of group lasso and minimum norm approaches to VEP source estimation.

### Single subject inversion

**Fig 3:**
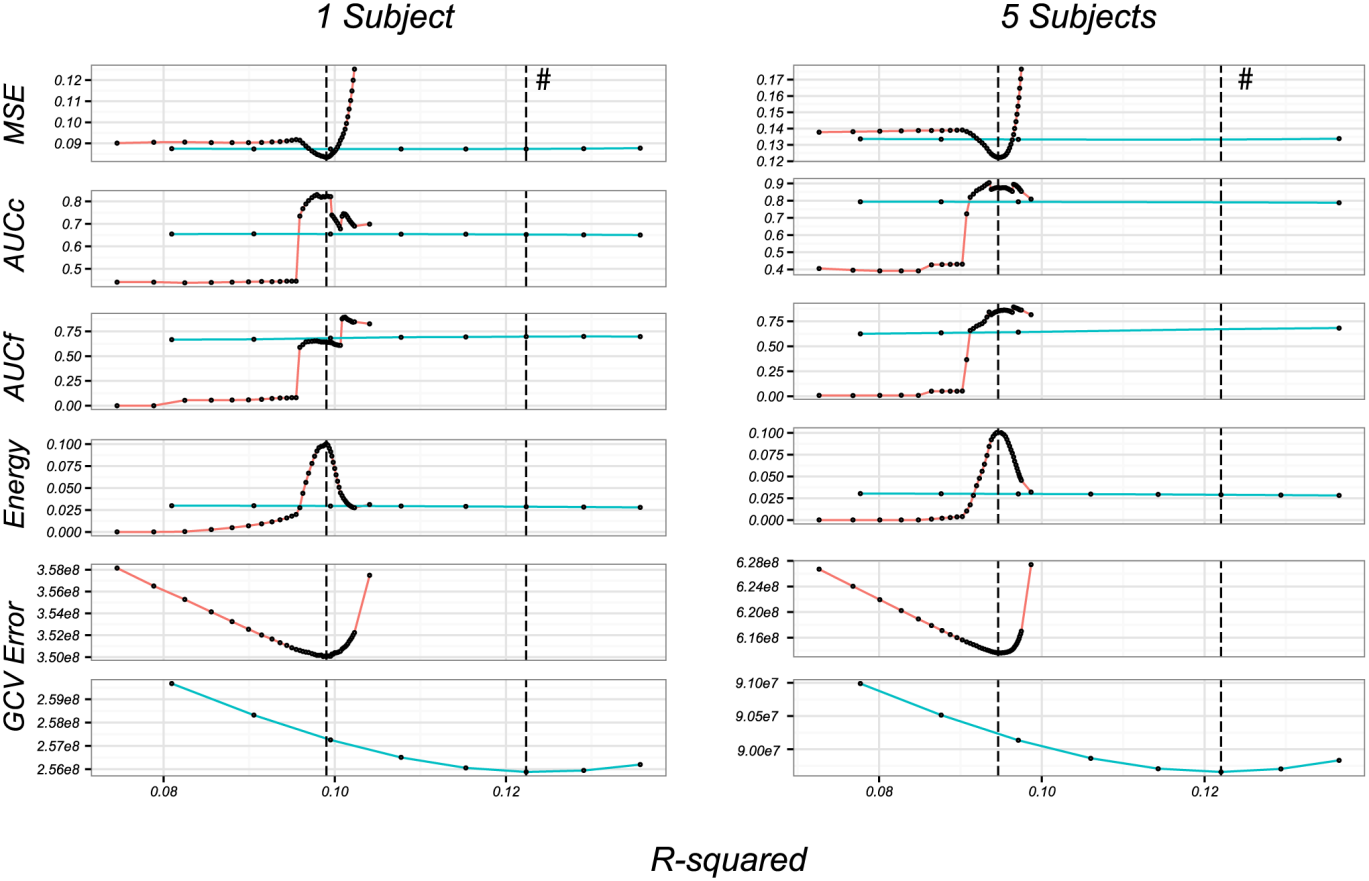
Performance of the group-lasso and minimum norm. Performance of the group-lasso (in red) and minimum-norm (in blue) on one instance of simulated data for one (left column) and five subjects (right column). Vertical lines correspond to the solutions chosen by optimizing the GCV error curve for each method, with the asterisks indicating the results from the minimum norm. The values obtained for the MSE, AUC close and far and energy and energy metrics are provided on the four first rows. Because there is no left and right subspace reduction with the minimum-norm, the GCV curve for this approach has a different scale than the one obtained with group-lasso. We therefore displays these curves separately on the fifth and sixth rows.

The performance of the group-lasso inversion method is first illustrated for the single subject case. Because both the group-lasso and minimum-norm methods produce a sequence of fits, we can visualize their performance as we move along their solution paths. One way to do this is to plot their performance as a function of fraction of variance explained (*r*^2^) on the training data. The *r*^2^ is defined by

$$\begin{array}{c} r^{2}=1-\frac{{∥Y-\hat{Y}∥}_{F}^{2}}{{∥Y-\bar{Y}∥}_{F}^{2}} \end{array}$$ (27)

where $\hat{Y}$ is the fit and $\bar{Y}$ is the matrix whose *i*-th column is the mean of the *i*-th column of **Y**. We plot the three metrics as a function of *r*^2^ in Fig 3. Curves are plotted against the *r*^2^ of the model fit on the training data rather than against *λ* in these figures. Since *r*^2^ is monotone increasing as *λ* decreases, they both give a measure of model complexity, but the former is more interpretable. We see that the group lasso outperforms the minimum norm on the AUC value and also on the relative energy. The mean-square errors (MSE) are comparable between the two algorithms. This is to be expected because there is no pooling effect for a single subject. The vertical dashed lines indicate the value of *λ* chosen by GCV (row five for group lasso, and six for minimum norm); these are shown separately since GCV is computed differently in each of these cases.

### Inversion over a groups of subjects

As noted in the introduction, variable selection can be made not only within an ROI of a given subject, but also across subjects and this is expected to result in improved estimates. To demonstrate this collaborative effect, we selected 5 subjects at random from our database and made the same comparison as in the previous section on a single instance of simulated data. These results are shown in Fig 3 (right column). While the results are qualitatively similar, notice that the group lasso does better than in the single subject case. In particular, there is a greater improvement relative to minimum norm in MSE (row 1), as well as in near and far AUC values (rows 2 and 3). The group model is able to assimilate information across subjects to decide if an ROI should be activated or not. The minimum-norm solution does not aggregate information across the multiple subjects, so that its performance remains similar to the single subject case.

**Fig 4:**
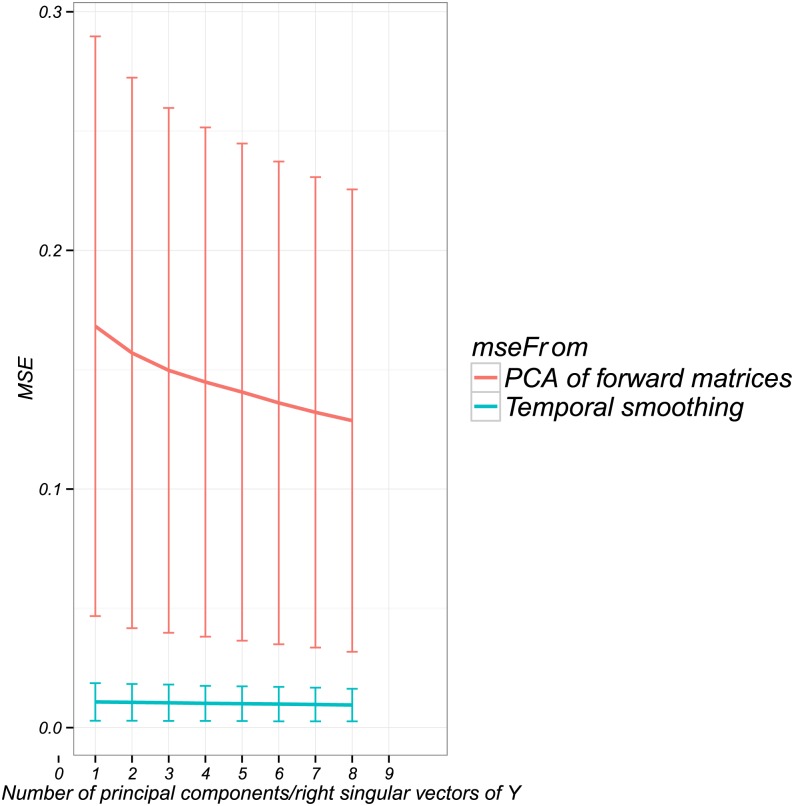
MSE from dimension reduction by principal components (in red) and temporal smoothing (in blue) with right singular vectors of Y, averaged over 100 simulations. A large portion of the MSE in our model is due to the dimension reduction from taking the first 5 principal components for each ROI, and a negligible portion is due to the temporal smoothing.

The minimum MSE for the group-lasso solution (in both the 1 and 5 subject cases) is around 0.1. We investigate the minimal attainable MSE in light of our temporal and spatial smoothing in Fig 4. To see this, we generate activity ***β*** for a single subject, then compute

$$\begin{array}{c} {∥\beta -PP^{T}\beta ∥}_{F}^{2} \end{array}$$ (28)

for varying numbers of principal components, and

$$\begin{array}{c} {∥\beta -\beta V_{d}V_{d}^{T}∥}_{F}^{2} \end{array}$$ (29)

for varying *d* (**P** and **V**_*d*_ defined in Eqs (8) and (17)). These computations tell us how we can expect to perform on MSE if we knew the true activity ***β***, but subjected it to the smoothness constraints. The true MSE in Fig 4 is of the same order as that seen in Fig 3 (third row). We see that the spatial smoothing is the limiting factor in this case, not the temporal smoothing. The rapid increase in MSE (red curves in first row of Fig 3) is due to an increase in variance as we decrease the amount of regularization. For a single subject, there are *Nd* observations and 18 ⋅ 5 ⋅ *d* parameters (see above Eq (19)), so that as *λ* ↓ 0, we approach a near-saturated fit.

**Fig 5:**
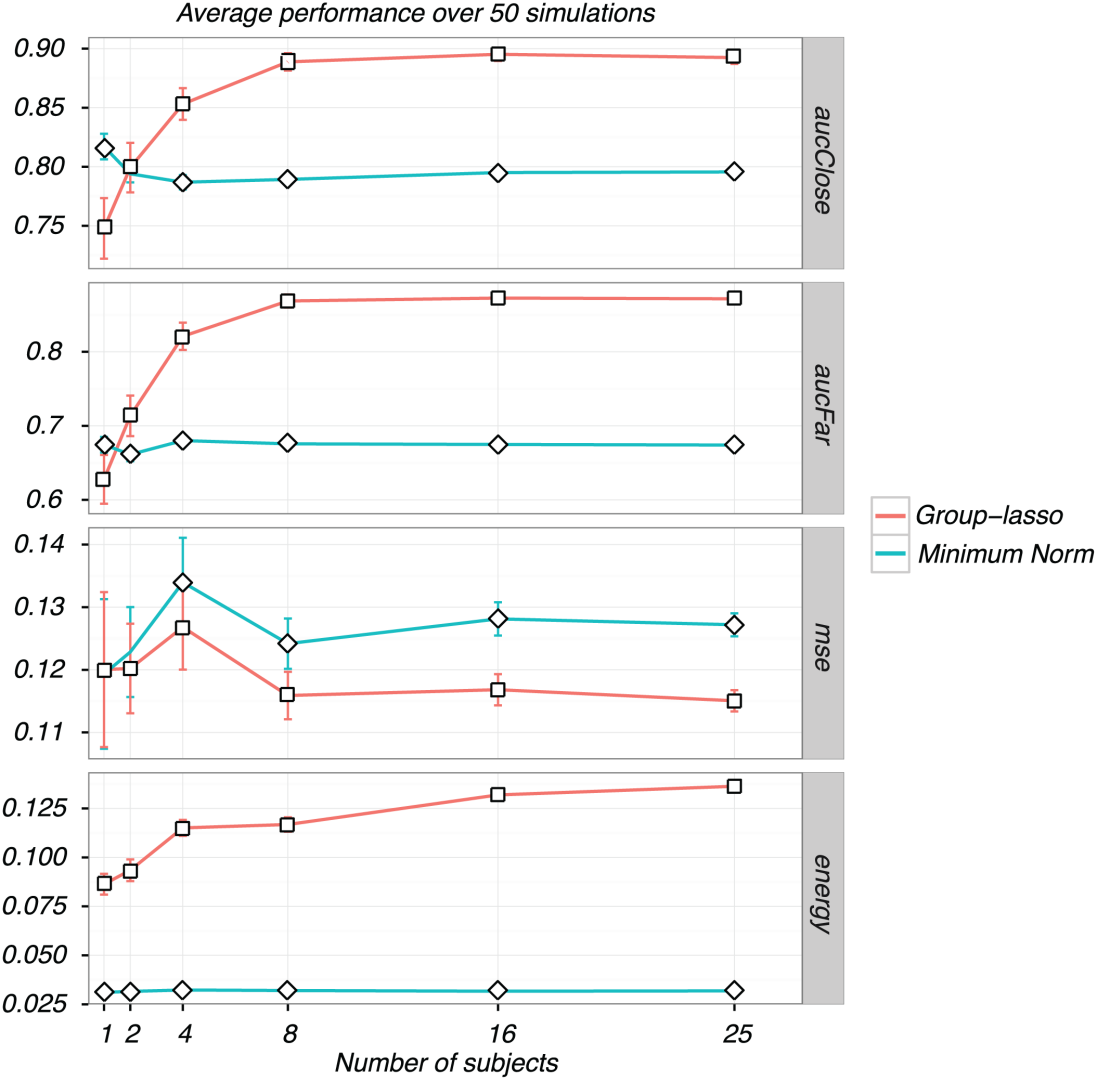
Performance of the group-lasso and minimum norma as a function of the number of subjects. Performance of the group-lasso is shown in red and the minimum norm in blue as a function of the number of subjects. Plots are of averages from 50 simulations with different subsets of subjects. Vertical lines are standard error bars. The group lasso performance improves with increasing numbers of subjects for the AUC and energy metric, but the minimum norm does not. MSE does not vary systematically with number of subjects for either inverse type.

We then repeated the analysis for successively larger groups of subjects, and with results averaged over 50 independent simulation runs. The average metrics (each chosen by GCV as in Fig 3), along with standard error bars, are plotted as a function of the number of subjects in Fig 5. As before, the minimum-norm solution does not pool information across multiple subjects, so that the performance stays flat despite having more subjects. The group-lasso clearly benefits from having more subjects, but this benefit tapers off after about 8 subjects. Also as before, the MSE does not improve for either of them as the number of subjects increases, but rather levels off. Again, bias is the limiting factor in both cases. For the group-lasso, the ROI-specific spatial bias is determined by the number of spatial principal components used (see Fig 4). The dip in MSE for two subjects is probably just noise (the error bars are wider here).

### Benefits of ROI-based, cross-subject averaging

**Fig 6:**
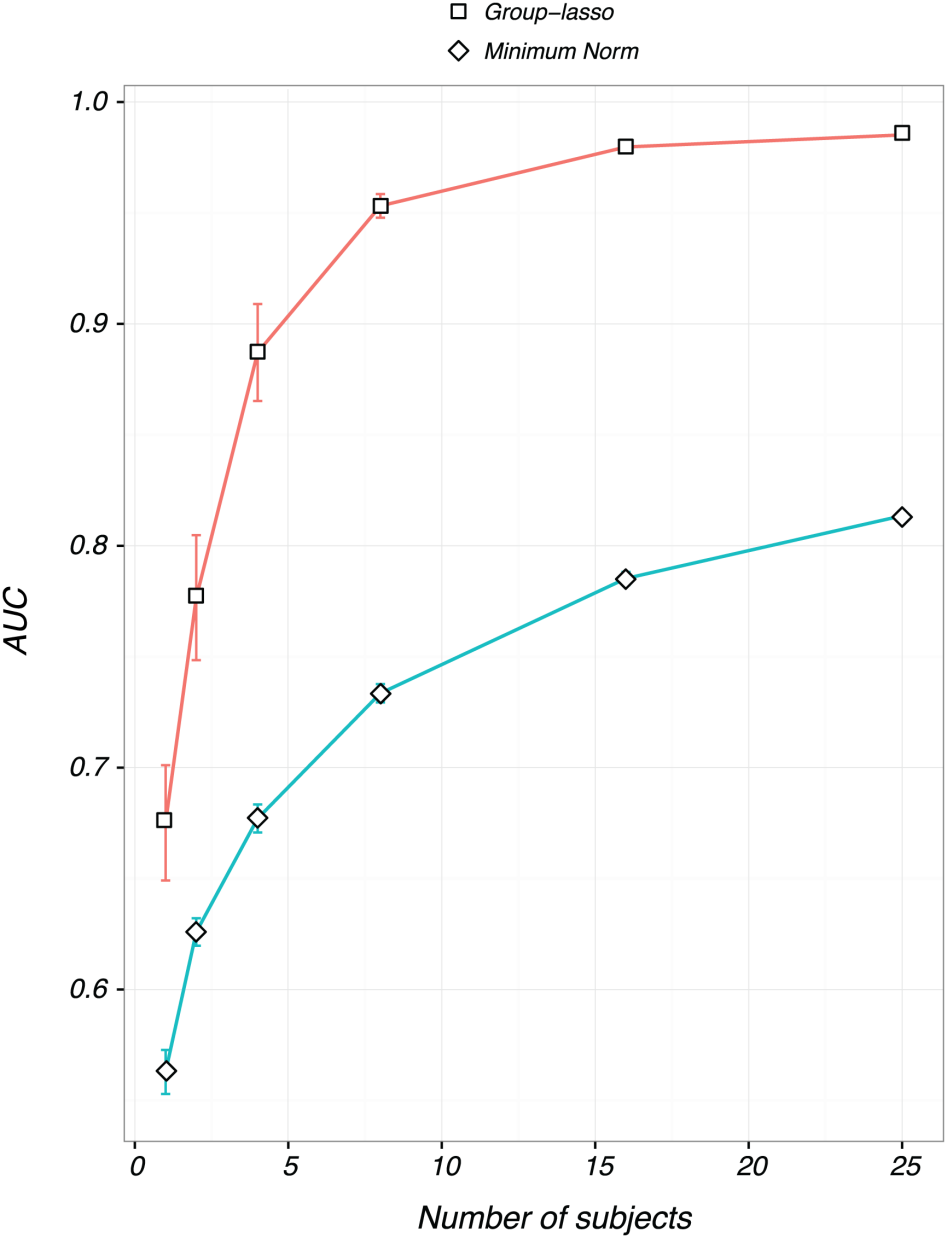
AUC obtained after post-processing the recovered activity by averaging across subjects. Plots are of average values over the same 50 data instances from before, along with standard error bars. Notice that the group-lasso with 4 subjects often outperforms the minimum norm with 25 subjects. Minimum norm in blue, group lasso in red.

The availability of ROI-based source estimates provides a functionally meaningful common space for cross-subject averaging of source estimates. A particular benefit of this approach is that the full 3-D structure of each ROI is implicit in the averaging process. Cross-talk projections into the common ROI from other active sources in other ROIs will tend to cancel in this form of averaging, while the activity within the target ROI will be “coherent” and survive the averaging post-process. We again studied how the performance of the group-lasso and minimum norm inverse methods scale with the number of subjects. We took 1, 2, 4, 8, 16, and 25 subjects, and for each situation, we fit the group-lasso and minimum-norm methods on 50 different instances of simulated data. We then computed the performance across the 50 simulations on the basis of the cross-subject average activations recovered by each method. The results are shown in Fig 6. Note that here the AUC calculation is based on classifying the activity of whole ROIs, rather than individual vertices.

Both methods get a substantial boost from ROI-based averaging across subjects, and performance improves as the number of subjects is increased. This is, we believe, a novel result, and is distinct from with the effect shown in Fig 5. There, the performance gain is due to the “majority vote” mechanism of the group-lasso as illustrated in Fig 1. Post-processing the recovered activity by ROI averaging serves to further reduce the variance in the estimates, thus resulting in a higher AUC for not just the group-lasso, but also for the minimum norm.

### Evaluation of group lasso solution on human VEP data

**Fig 7:**
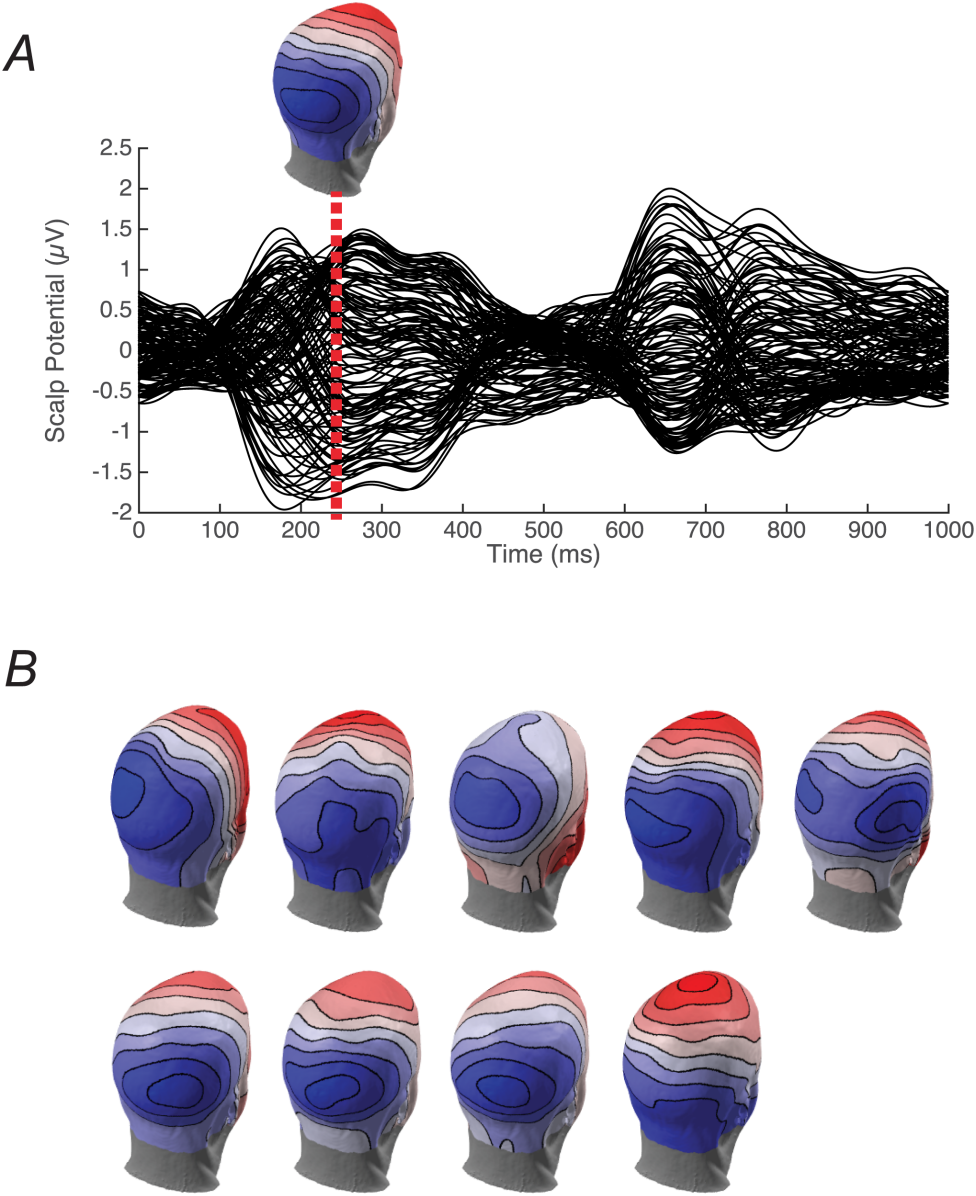
SSVEP to alternations between coherent and incoherent motion. Panel A is the group-average (n = 9) waveforms from all 128 EEG sensors. The inset shows the group-average scalp topography. Panel B shows the individual participant topographies for all 9 participants that went into to group average. The topography is shown for the same time as in panel A.

To compare the performance of the group lasso and minimum norm solutions on real data, we chose to use a coherent motion visual stimulation paradigm. This choice was motivated by the fact that the underlying sources of the coherent motion response have been studied extensively using fMRI [58–61]. These studies consistently show the strongest activations to be in the human MT complex and in V3A when the contrast is made between coherent and incoherent motion. The expected activation is thus rather sparse among the visual ROIs we have used in the simulations. An SSVEP paradigm was used that creates data with a high signal-to-noise ratio [62]. This paradigm periodically exchanges coherent and incoherent motion and the resulting response can be interpreted as arising from areas that can discriminate the two types of motion, analogous to an fMRI contrast. The group-average sensor data has a complex waveform (Fig 7A). The first 500 ms reflects the evoked response to the onset of coherent motion, while the second 500 ms corresponds to its offset (from coherent to incoherent or random motion). Coherent motion onset at 0 msec creates a clear occipital focused topography, illustrated for the group data at 250 msec (Fig 7A, inset).

**Fig 8:**
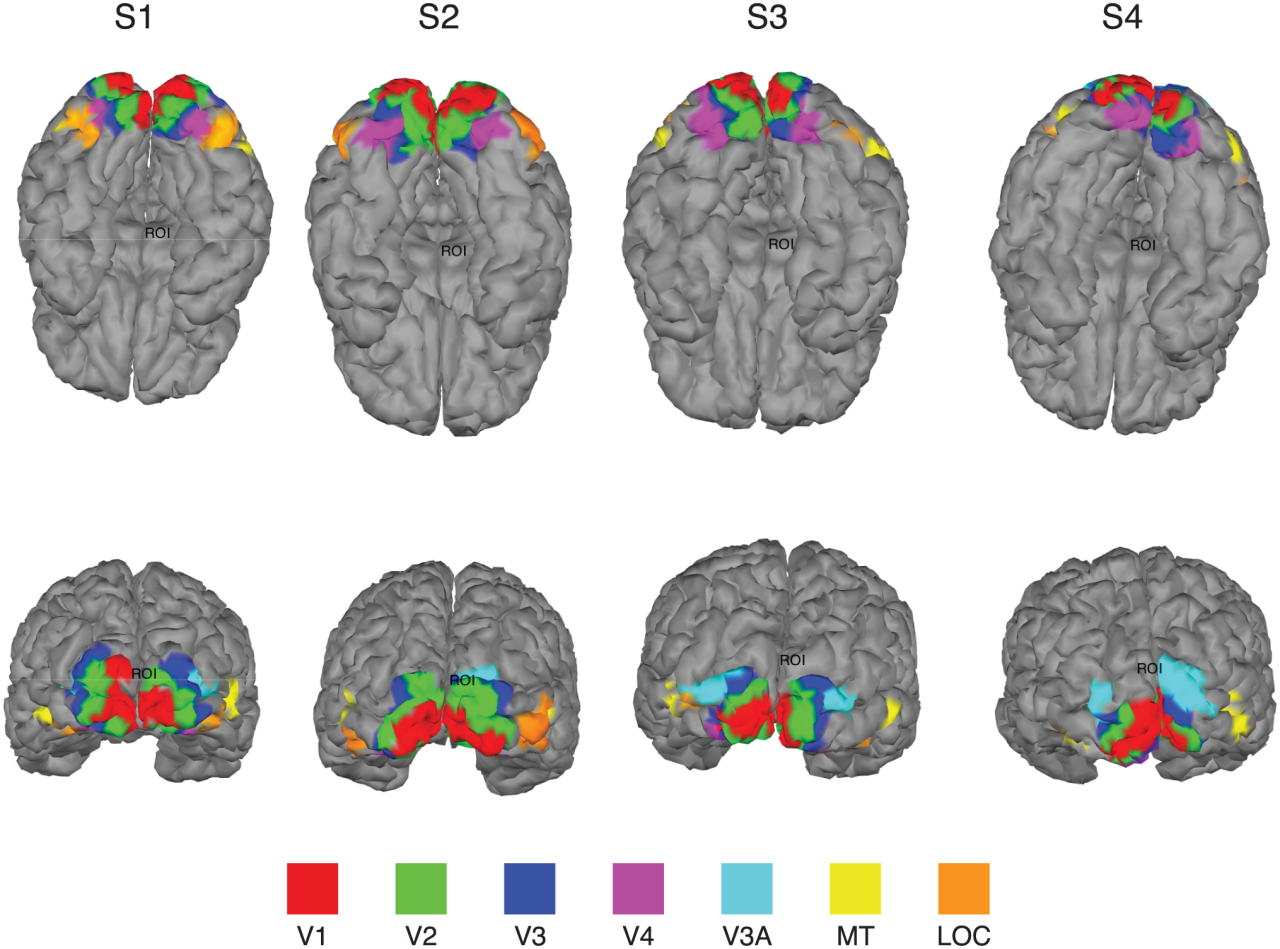
Representative cortical surface reconstructions. Visual ROIs V1, V2, V3, V3A, V4, MT and LOC are shown (see color bar for labeling convention). Top panel shows ventral surface view, bottom panel posterior view. Note that while there is a general pattern of agreement in the relative location of the visual areas, there is considerable variability in the detailed shape, size and location of the ROIs across subjects.

Note however that the group-averaged topography masks substantial, cross-participant differences (Fig 7, panel B). These differences presumably arise from individual differences in location and 3D shape of the visual areas activated by coherent motion. Examples of the cross-subject variability of visual areas in size, shape and location are shown in Fig 8 for four subjects. Recall that several of the areas were defined on the basis of retinotopic mapping (V1, V2, V3, V4, V3A), but two (MT and LOC) were defined by functional localizers. Note that for each area there is a general consensus as to the location of the area, but the details of the shape and neighbor relationships are idiosyncratic. The group lasso on ROI-based features takes advantage of these differences to create more focal source estimates.

**Fig 9:**
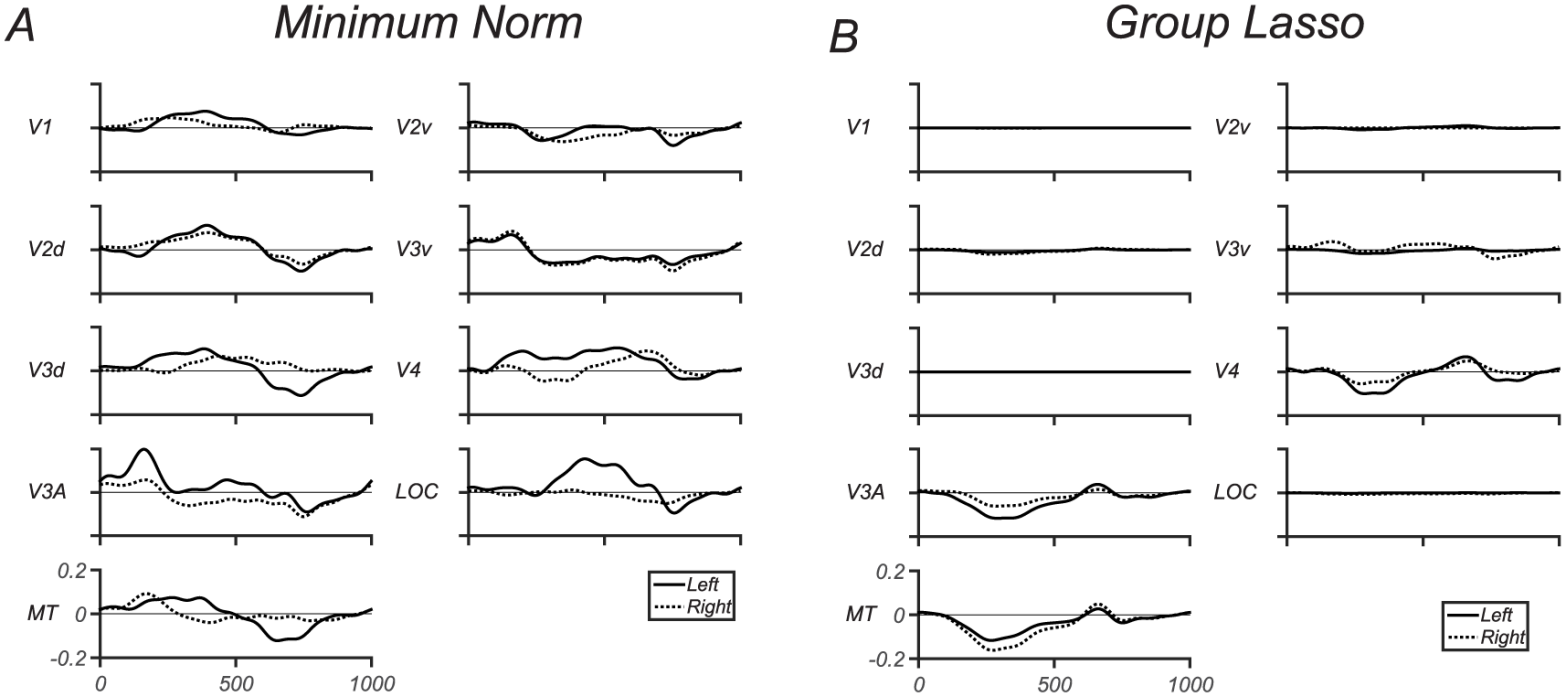
(A) Minimum norm solutions for coherent/incoherent motion SSVEP responses in visual ROIs. With the minimum norm all visual ROIs contain some level of activation. With left and right ROIs showing differences. (B) group Lasso solutions for coherent motion SSVEP responses in visual ROIs. With the group Lasso only a few of the visual ROIs contain some level of activation. With left and right ROIs showing similar waveforms. Group lasso solution produces stronger distinctions between MT and V3A ROI activations and the other ROIs than does the minimum norm.

Evoked response time-courses for the coherent motion task are shown for this set of ROIs in Fig 9 both the minimum norm (panel A) and lasso (panel B) solutions. The ROI time-courses for the minimum norm solution show the response to be distributed widely across the visual ROIs (Fig 9A). In addition, several ROIs show marked differences between left and right hemisphere time-courses. Because the visual stimulus was large and viewed centrally, it is expected to generate a mostly symmetric activation of the left and right hemispheres. By contrast, the group-lasso solution (Fig 9B) shows clearer distinctions between areas, with the largest responses in V3A and hMT+, as expected from prior work in human fMRI with similar stimuli [58–61]. As noted above, the activations are similar between the two hemispheres, consistent with the large field stimulus. The most dramatic difference between inverses occurs for the activations from the LOC and hMT+. These ROIs are physically adjacent in cortex, however LOC responds to objects, while MT responds to motion [58–61]. The minimum norm spreads activation over both ROIs while the group Lasso provides a strongly active MT and silences the LOC.

**Fig 10:**
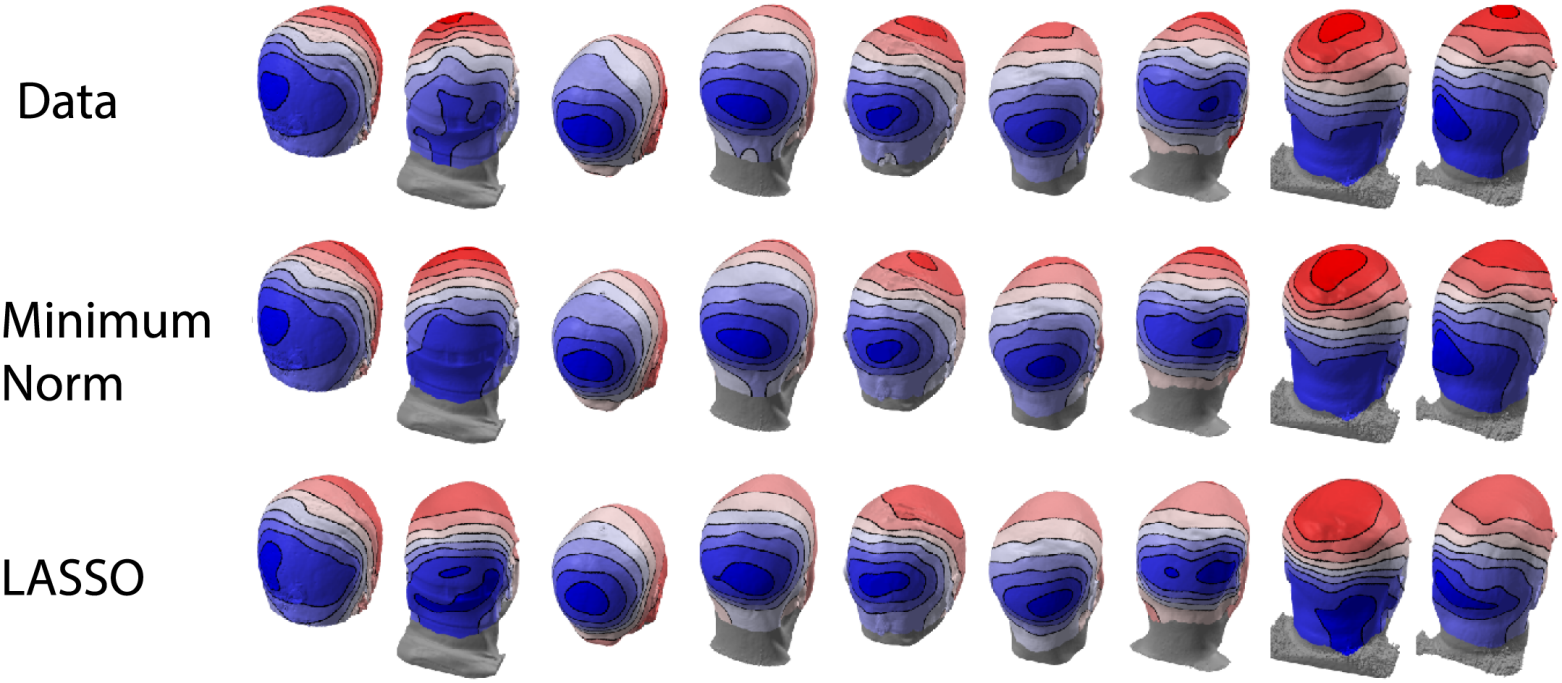
Scalp topographies from measured data and reconstructed from inverse solutions. (Top) The top row is the original SSVEP data demonstrating cross-participant heterogeniety. (Middle) Reconstructed topographies from the minimum norm solution. (Bottom) Reconstructed topographies from the group Lasso solution. Even though the group lasso solution utilizes fewer cortical areas than the minimum norm it is still able to capture the cross-participant heterogeniety.

Both the minimum norm and the group-lasso are able to account for the cross-participant differences in the topographic data (Fig 10). Estimates of the sensor-space data at a single time-point (250 msec) were generated by projecting the source-space activations from each inverse solution through each subject’s forward matrix. Importantly, the group-lasso can fit the sensor-space data with activations from only three ROIs, but the minimum norm solution requires all ROIs to be active. Consistent with the results of the simulations, these tests on real data confirm that group lasso outperforms the minimum norm and is able to recover a sparse set of underlying activations.

## Discussion

We have introduced a new approach to EEG/MEG source estimation—the group lasso—that provides a type of sparse-inversion procedure. In agreement with previous studies that have compared *ℓ*_1_ *vs* *ℓ*_2_ (minimum-norm) inverse procedures [19, 20, 23, 28], the group lasso out-performs the minimum-norm procedure. Here performance was quantified in terms of AUC and focality measures derived from a simulation that used sources that are a realistic representation of sources that are expected to be active in visual processing tasks. Separating sources via inverse methods is particularly difficult in the visual system because the ROIs can be in close spatial proximity and because of the complexities that result from folding and positioning of the surface of the brain with respect to itself and the sensors. In general, these effects cause some regions to be aliased with others in the inverse, effectively competing with each other in claiming responsibility for the signal. The group-lasso procedure provides a natural way of introducing prior knowledge about the sources—derived from independent MRI and fMRI measurements—that can be incorporated as constraints on the inversion process. These constraints are relevant to the related problems of feature selection, the smoothing of sparse-inverse solutions and finding a common space for estimating sources in groups of participants. Our approach offers a direct and simple way to tie together activity in multiple subjects via their ROI activity, *without* having to warp each brain to a common source space, with the attendant spatial distortion. This is in contrast to the existing hierarchical-Bayes approaches [15, 35] which do require this common warping. We also avoid the considerable complexity of having to work with high-dimensional structured covariance matrices.

### Feature selection via the group-lasso

As noted, the ROIs we use provide a natural means of grouping features both within a given subject (the vertices within an ROI constitute a group) and across subjects (the penalty enforces consistency across subjects for the activation level of a given ROI, a second level of feature grouping). In the first case, because functional areas have consistent selectivity within an area and possibly different selectivity between areas, it is natural to group vertices of the cortical mesh on the basis of which ROI they belong to. Secondly, because it is a reasonable assumption that a given ROI has the same functional selectivity across subjects, it is natural to enforce group-consistency on this basis, as well. Through this constraint, the group lasso is able to pool information across multiple subjects in a way that improves the source estimates for individual subjects. In our case, a group is the union of the vertices in the corresponding ROIs across the subjects. Recall that the same ROI can have different orientations from subject to subject, so that in some subjects, a ROI might have weak explanatory power for the signal (due to cancellation or correlation with other ROIs), but this same ROI could be strong in other subjects. The group lasso “settles disputes” by giving the responsibility to the region that appears to be strongest in aggregate over all the subjects. The group-lasso thus estimates the sources for an entire ROI to be zero or nonzero, providing a focal estimate of which areas are responsible for generating the signal.

### Smoothing in the context of ROI-based groups

Basing the group selection on functional ROI’s also provides a meaningful way of enforcing spatial smoothness (within a group/functional ROI) without the over-smoothing across functional boundaries that would occur with simple near-neighbor smoothing approaches such as that used in LORETA [5]. ROI-based parcellation retains the advantages of previous patch-based parcellation approaches [57, 63, 64] but makes them more precise through an independent measurement of the extent and borders of retinotopic maps and functional areas. In retinotopic areas, neighboring locations within an area have correlated activity because the point-to-point nature of the mapping from visual space onto cortex creates strong neighborhood relationships, particularly for extended stimuli. In addition, neighborhood correlations are created within areas via lateral and feedback connections [65]. Functionally defined areas, such as the LOC are also likely to show correlated activity due to the fact that they encode stimuli of a common class over considerable regions of visual space [66]. These natural occurrences of correlated activity in visual areas provide useful prior information on the source-covariance matrix: e.g. which locations should be more or less correlated because of the location with respect to a visual field map or a functional area. These correlations provide a further rationale for the use of group variable selection via the lasso both within an individual subject’s source space and across participants.

### Benefits of functional ROI’s as the common space for inversion

The location of visual areas is partially constrained to sulci and gyri, with the relationship being tighter in V1 and V2, located in and around the calcarine sulcus, and looser in higher-order extra-striate areas [67]. Fortunately, the 3D shapes and locations of these areas can be measured accurately in individual participants by a combination of functional and structural MRI. Here, the lead field is formed on the basis of accurate 3D surface normals of the vertices that have been identified as belonging to a given functional area. Our group-lasso method takes advantage of the cortical surface normals when averaging across subjects. Surface normals are not well-preserved preserved by volume-based template procedures to common spaces for inversion. Because fMRI mapping is expensive and time-consuming, a viable alternative to individual mapping of topographic ROI is to use atlas-based procedures that do retain surface normals [68–70]. The utility of surface based atlas procedures will depend on the area to be mapped, as the quality of the atlas fit to a given individual varies as one goes from early visual areas in calcarine cortex to higher-order extra-striate areas. Outside of the visual system, group-lasso could be by using anatomically or functionally informed ROIs. For example, a recent atlas that is based on multiple functional and structural criteria could be used [70]. This atlas defines 180 cortical areas in each hemisphere. Here again, the accuracy of source localization will depend on the accuracy of the atlas used, but we expect that this approach will still be advantageous.

### Two independent benefits from cross-subject averaging

We also showed how to combine data from multiple subjects while also imposing spatial and temporal smoothness on the recovered activity as provided by the ROIs forming the groups. The “pooling effect” of the group-lasso suggests that its performance should improve with the number of subjects, and we verified this with simulation experiments. In particular, while performance of the group-lasso is roughly comparable to that of the traditional minimum-norm solution for a single subject, as the number of subjects increases, there is a significant performance increase for the group-lasso over the minimum norm. This occurs because the estimate of an individual subjects activations is facilitated by enforcing consistency across the group of subjects. The minimum norm and previous sparse-inverse methods do not inherently pool information across multiple subjects. Once the ROI activations have been determined for a given participant, further, independent improvements can be achieved by averaging these estimates across subjects. This averaging effect applies to both the ROI-based minimum norm procedure and the group-lasso solution and is quite substantial [33].

### Limitations of the present study

A limitation of simulation studies is the accuracy with which the simulated data reflects the data that are likely to occur in practice. Here we took advantage of our prior knowledge of the functional organization of the visual cortex to create simulation data. To the extent that visual stimuli primarily activate these areas, the simulation is reasonably realistic. We included variations in the extent to which a given area is active, as well. We were able to verify the superiority of the lasso method over the minimum norm for a small data set from a visual activation study using a comparable set of conditions and common space. Nonetheless, future work with a wider range of realistic activations where strong ground truth data, such as that from retinotopic stimulation protocols are available would be useful. Simulation studies are likely to overestimate performance in practice due to the use of the same head model for forward and inverse calculations. However these errors will affect both methods we evaluated, leaving the relative comparisons valid.

It will also be of use to compare the present method with other methods. Such comparisons are not trivial, given that different approaches as used in practice differ not only in the inversion algorithm itself, but also in the assumed common space, the nature of the head model and other factors that would need to be held constant to isolate the effect of the inversion procedure per se. In our approach, the ROI common space is very strongly embedded not only in feature selection via grouping, but also in the second stage of cross-subject averaging. One would thus want to separately evaluate the contribution of choice of common space by using realistic ROI’s with other inversion algorithms instead of using warping to a common space, arbitrary parcellations [57, 63, 64] or smoothing functions [5]. As noted above a comparison of inversion methods that retain surface normals, such as ours or [71] for example, compared to methods that don’t would be of interest. For methods that treat source activity as an unsigned scalar quantity at a given location, we expect there to be little benefit on the AUC metric for averaging over increasing numbers of subjects as cross-talk between areas will all have a common “polarity” rather than positive and negative polarities that will partially cancel when averaged across mulitple subjects [33]. Finally a comparison of our norm-based approach to hierarchical Bayesian approaches done within the same common space would be of interest [10–13]; (see [14] and [15] for reviews).

### Future directions

The effectiveness of the group-lasso can lead to other interesting possibilities. The overlapped group-lasso is a special case of the group-lasso in which a variable can show up in more than one group. It follows that the overlapped group-lasso might be a good choice for source inversion in cases where the ROIs have overlaps. Another aspect of ROI-wise source inversion that we have not explored is sparsity within a ROI. It is possible that the source activity is only present in some fraction of the ROI, so that a solution that is sparse within a ROI is desirable. If this is the case, an additional *ℓ*_1_ penalty of the form ‖***β***‖_1_ can be added to the group-lasso penalty to impose sparsity. This results in what is known as the sparse-group lasso (see [72] for details). This approach, along with the overlapped group-lasso, will likely be fruitful lines for further investigation.

## Supporting information

S1 Model Selection Details (PDF)

S1 FigEstimated degrees of freedom (using (S1-1)) vs true df.Red line: Using formula (S1-1) without any ridge penalty to ${\hat{\beta }}^{0}$ results in an estimate that is biased downward. Blue line: In our experiments, a ridge penalty of 1.0817 × 10^4^ works well.(EPS)

S2 FigVariance of ${\hat{\beta }}^{0}$ as a function of ridge parameter.Vertical line corresponds to 1.0817 × 10^4^ that is found to work well in our degrees of freedom simulations.(EPS)

S1 Algorithm Details (PDF)
