# Supplementary material for: Sparse EEG/MEG source estimation via a group lasso
Source: PLoS One. 2017 Jun 12;12(6):e0176835. doi: 10.1371/journal.pone.0176835 (PMC5467834; doi:10.1371/journal.pone.0176835)
Supplement: S1 Model Selection Details — (PDF) [file pone.0176835.s001.pdf]

S1 Model Selection Details

To the best of our knowledge, there is no analytic form for the degrees of freedom for the group-lasso. We use the results in [1] as a heuristic for estimating the degree of freedom. Suppose there are G groups of variables with sizes $p_1, \ldots, p_G$. Let $\hat{\boldsymbol{\beta}}^0$ denote the full ordinary least squares fit, $\hat{\boldsymbol{\beta}}_i$ the group-lasso estimate for group i, and $\hat{\mathbf{Y}}$ the fit. Then if the feature matrix $\mathbf{X} = [\mathbf{X}_1, \ldots, \mathbf{X}_G]$ is orthonormal, an unbiased estimate of the degrees of freedom is given by

$$df(\hat{\mathbf{Y}}) = \sum_{i=1}^{G} \mathbf{1}(\|\hat{\boldsymbol{\beta}}_i\|_2 > 0) + \sum_{i=1}^{G} (p_i - 1) \frac{\|\hat{\boldsymbol{\beta}}_i\|_2}{\|\hat{\boldsymbol{\beta}}_i^0\|_2}. \tag{S1-1}$$

Because $\mathbf{X}$ is not orthonormal in our application, we do not expect this formula to hold exactly. Simulations show that the estimate given by (S1-1) can be biased low, which results in an overly optimistic GCV error rate. The bias is due to large variance in $\hat{\boldsymbol{\beta}}^0$, making the contribution from the $\frac{\|\hat{\boldsymbol{\beta}}_i\|_2}{\|\hat{\boldsymbol{\beta}}_i^0\|_2}$ term negligible. Adding a ridge penalty (our α) when computing $\hat{\boldsymbol{\beta}}^0$ largely eliminates this problem. To verify this, we generate data according to the following setup.

Let $\mathbf{Y}_{fixed}$ be a fixed $N \times T$ matrix of observations. Assume $\mathbf{Y} = \mathbf{Y}_{fixed} + \boldsymbol{\epsilon}$ with $\boldsymbol{\epsilon}$ also $N \times T$, with iid elements each having mean 0 and variances σ^2. Then the degrees of freedom of a fit $\hat{\mathbf{Y}}$ is given by [2]

$$\begin{aligned}
\frac{1}{\sigma^2} \sum_{s=1}^{t} \sum_{i=1}^{N} \mathrm{Cov}(\mathbf{Y}_{is}, \hat{\mathbf{Y}}_{is}) &= \frac{1}{\sigma^2} \sum_{s=1}^{t} \mathrm{tr}\,\mathrm{Cov}(\mathbf{Y}_{\cdot s}, \hat{\mathbf{Y}}_{\cdot s}) && \text{(S1-2)} \\
&= \frac{1}{\sigma^2} \sum_{s=1}^{t} \mathrm{tr}\,\mathrm{Cov}(\boldsymbol{\epsilon}_{\cdot s}, \hat{\mathbf{Y}}_{\cdot s}) && \text{(S1-3)} \\
&= \frac{1}{\sigma^2} \sum_{s=1}^{t} \mathrm{tr}\,\mathbb{E}(\boldsymbol{\epsilon}_{\cdot s} \hat{\mathbf{Y}}_{\cdot s}^t) \qquad (\boldsymbol{\epsilon}_{\cdot s} \text{ has mean } 0) && \text{(S1-4)} \\
&= \frac{1}{\sigma^2} \sum_{s=1}^{t} \mathbb{E}\,\mathrm{tr}(\boldsymbol{\epsilon}_{\cdot s} \hat{\mathbf{Y}}_{\cdot s}^t) && \text{(S1-5)} \\
&= \frac{1}{\sigma^2} \sum_{s=1}^{t} \mathbb{E}(\hat{\mathbf{Y}}_{\cdot s}^t \boldsymbol{\epsilon}_{\cdot s}) \qquad \text{(trace of scalar is itself)} && \text{(S1-6)} \\
&= \frac{1}{\sigma^2} \mathbb{E}\,\mathrm{tr}(\hat{\mathbf{Y}}^t \boldsymbol{\epsilon}) && \text{(S1-7)}
\end{aligned}$$

It follows that we can estimate the true degrees of freedom by generating noisy observations $\mathbf{Y} = \mathbf{Y}_{fixed} + \boldsymbol{\epsilon}$ and averaging the trace of $\hat{\mathbf{Y}}^t \boldsymbol{\epsilon}$ over many simulations. We can then compare the results with the formula in (S1-1). S1 Fig 1 shows the results from 1000 simulations with $\sigma = 5$.

We have seen that adding a ridge penalty to the ordinary least squares fit in the degrees of freedom formula leads to a more stable and accurate estimate. Because the group-lasso estimates converge to the ordinary least squares estimates (for "$p < n$" problems) as $\lambda \downarrow 0$, we use the same amount of ridging by setting $\alpha = 1.0817 \times 10^4$ in (7); see the effect on the variance in S1 Fig 2. This is the value we used in all our experiments.

S1 Fig 1. Estimated degrees of freedom using (S1-1) vs true df. Red line: Using formula (S1-1) without any ridge penalty to $\hat{\boldsymbol{\beta}}^0$ results in an estimate that is biased downward. Blue line: In our experiments, a ridge penalty of 1.0817×10^4 works well.

S1 Fig 2. Variance of $\hat{\boldsymbol{\beta}}^0$ as a function of ridge parameter. Vertical line corresponds to 1.0817×10^4 that is found to work well in our degrees of freedom simulations.

References

1. Kato K (2009) On the degrees of freedom in shrinkage estimation. Journal of Multivariate Analysis 100 (7): 1338–1352.

2. Efron B (1986) How Biased is the Apparent Error Rate of a Prediction Rule? Journal of the American Statistical Association 81: 461–470.
