# Supplementary material for: Sparse EEG/MEG source estimation via a group lasso
Source: PLoS One. 2017 Jun 12;12(6):e0176835. doi: 10.1371/journal.pone.0176835 (PMC5467834; doi:10.1371/journal.pone.0176835)
Supplement: S1 Algorithm Details — (PDF) [file pone.0176835.s004.pdf]

S2 Algorithm Details

We describe the algorithm used to obtain the solutions to (7). Cyclic group-wise coordinate descent works well when we have a small number of groups (18 ROIs in our case). We compute a family of solutions varying λ from λ_{max} down toward zero over a grid of values (on the exponential scale), with λ_{max} being the smallest value of λ for which all the estimated $\tilde{\boldsymbol{\beta}}_i$ are zero [see equation (19)]. As) we move down the λ sequence, we use "warm starts" from the previous value of λ to start the iterations. The idea of coordinate descent is to update the coefficients for a single group while holding the coefficients for all other groups fixed. If we cycle through all the groups repeatedly, we will converge to the solution of the strictly convex optimization problem at each λ [1]. Let $\mathbf{Y}$ be the matrix of observations, and let $\mathbf{X} = [\mathbf{X}_1, \ldots, \mathbf{X}_p]$ be the matrix of features that constitute the p groups.

S2.1 Cyclic group-wise coordinate descent

We consider the generic problem

$$\text{argmin}_{\boldsymbol{\mu},\boldsymbol{\beta}} \frac{1}{2} \left\| \mathbf{Y} - \mathbf{1}\boldsymbol{\mu}^t - \sum_{i=1}^{p} \mathbf{X}_i \boldsymbol{\beta}_i \right\|_F^2 + \lambda \sum_{i=1}^{p} \gamma_i \|\boldsymbol{\beta}_i\|_F + \alpha \|\boldsymbol{\beta}\|_F^2. \tag{S2-1}$$

We have introduced penalty modifiers γ_i, so in fact the regularization multiplier for group i is $\lambda\gamma_i$. These are needed to allow for potentially different group sizes and scales, and are discussed in S2.2. We first elliminate $\boldsymbol{\mu}$ by partially optimizing (S2-1) with respect to $\boldsymbol{\mu}$. It is easy to show that centering each of the columns of $\mathbf{X}_i$ is a simple re-parametrization of the problem that leaves $\boldsymbol{\beta}_i$ alone, but changes $\boldsymbol{\mu}$, and importantly leads to exactly the same optimized fit. But with these transformations, $\hat{\boldsymbol{\mu}}$ is given by the column means of $\mathbf{Y}$. With this $\hat{\boldsymbol{\mu}}$ we can replace $\mathbf{Y}$ by its centered version, and remove $\boldsymbol{\mu}$ from (S2-1), as long as we use the centered $\mathbf{X}_i$.

Let λ and α be fixed, and suppose we want to perform the update for group k. Let $\hat{\boldsymbol{\beta}}_1, \ldots, \hat{\boldsymbol{\beta}}_p$ be the current estimates, and define the partial residual for group k to be $\mathbf{r}_k = \mathbf{Y} - \sum_{i \neq k}^{p} \mathbf{X}_i \hat{\boldsymbol{\beta}}_i$ (i.e. in (S2-1), we have created a uni-block problem.) Using standard results in convex optimization, we write the subgradient equation for $\boldsymbol{\beta}_k$ in (S2-1):

$$-\mathbf{X}_k^t \mathbf{r}_k + \mathbf{X}_k^t \mathbf{X}_k \boldsymbol{\beta}_k + \lambda \gamma_k \mathbf{s}_k + 2\alpha \boldsymbol{\beta}_k = \mathbf{0}, \tag{S2-2}$$

where $\mathbf{s}_k \in \{\mathbf{z} : \|\mathbf{z}\|_F \leq 1\}$, and $\mathbf{s}_k = \boldsymbol{\beta}_k / \|\boldsymbol{\beta}_k\|_F$ if $\boldsymbol{\beta}_k \neq \mathbf{0}$. It follows that $\hat{\boldsymbol{\beta}}_k = 0$ if $\|\mathbf{X}_k^t \mathbf{r}_k\|_F < \lambda\gamma_k$. If $\hat{\boldsymbol{\beta}}_k \neq 0$, from (S2-2) we have

$$\left(\mathbf{X}_k^t \mathbf{X}_k + \left(\frac{\lambda \gamma_k}{\|\hat{\boldsymbol{\beta}}_k\|_F} + 2\alpha \right) \mathbf{I} \right) \hat{\boldsymbol{\beta}}_k = \mathbf{X}_k^t \mathbf{r}_k \tag{S2-3}$$

We can solve for $\hat{\boldsymbol{\beta}}_k$ by first solving for the scalar $\|\hat{\boldsymbol{\beta}}_k\|_F$ and then plugging the result into (S2-3) to get a closed-form solution.

We compute the singular value decomposition (one-time computation) $\mathbf{X}_k = \mathbf{U}_k \mathbf{D}_k \mathbf{V}_k^t$ and rewrite (S2-3) as

$$\left[\mathbf{D}_k^2 \|\hat{\boldsymbol{\beta}}_k\|_F + (\lambda\gamma_k + 2\alpha \|\hat{\boldsymbol{\beta}}_k\|_F) \mathbf{I} \right]^{-1} \mathbf{D}_k \mathbf{U}_k^t \mathbf{r}_k = \mathbf{V}_k^t \frac{\hat{\boldsymbol{\beta}}_k}{\|\hat{\boldsymbol{\beta}}_k\|_F}. \tag{S2-4}$$

Take the Frobenius norm on both sides to obtain

$$\left\| \left[\mathbf{D}_k^2 \|\hat{\boldsymbol{\beta}}_k\|_F + (\lambda\gamma_k + 2\alpha\|\hat{\boldsymbol{\beta}}_k\|_F)\mathbf{I} \right]^{-1} \mathbf{D}_k \mathbf{U}_k^t \mathbf{r}_k \right\|_F^2 = 1. \tag{S2-5}$$

Let $f(\theta) = \left\|[\mathbf{D}_k^2\theta + (\lambda\gamma_k + 2\alpha\theta)\mathbf{I}]^{-1}\mathbf{D}_k\mathbf{U}_k^t\mathbf{r}_k\right\|_F^2 - 1$. To find $\|\hat{\boldsymbol{\beta}}_k\|_F$, we need only find θ_0 such that $f(\theta_0) = 0$. We do this with Newton-Rhapson by reiterating

$$\theta \longleftarrow \theta - \eta \frac{f(\theta)}{f'(\theta)}, \tag{S2-6}$$

where η is the step size and

$$f'(\theta) = -2 \left\| \left[\mathbf{D}_k^2\theta + (\lambda\gamma_k + 2\alpha\theta)\mathbf{I} \right]^{-\frac{3}{2}} (\mathbf{D}_k^2 + 2\alpha\mathbf{I})^{\frac{1}{2}} \mathbf{D}_k \mathbf{U}_k^t \mathbf{r}_k \right\|_F^2. \tag{S2-7}$$

In our experience, $f(\theta)$ tends to be quite linear around θ_0, so that very few Newton iterations are required for convergence. Having obtained $\hat{\theta}_0$, we update $\hat{\boldsymbol{\beta}}_k$ with

$$\hat{\boldsymbol{\beta}}_k \longleftarrow \left(\mathbf{X}_k^t\mathbf{X}_k + (\frac{\lambda\gamma_k}{\hat{\theta}_0} + 2\alpha)\mathbf{I} \right)^{-1} \mathbf{X}_k^t\mathbf{r}_k. \tag{S2-8}$$

We now cycle through all the groups until convergence. The full algorithm is presented in Algorithm 1. In

Algorithm 1: Cyclic group-wise coordinate descent

input : $\mathbf{Y}$, $\mathbf{X}_1, \ldots, \mathbf{X}_p$, $\lambda, \gamma_1, \ldots, \gamma_p, \alpha$. All $\mathbf{X}_j$ and $\mathbf{Y}$ column centered.
output: $\hat{\boldsymbol{\beta}}_1, \ldots, \hat{\boldsymbol{\beta}}_p$

Initialize $\mathbf{r} = \mathbf{Y}$, $\hat{\boldsymbol{\beta}}_1 = 0, \ldots, \hat{\boldsymbol{\beta}}_p = 0$. Let $\mathbf{X}_k = \mathbf{U}_k\mathbf{D}_k\mathbf{V}_k^t$ be the singular decomposition of $\mathbf{X}_k$.

Iterate until convergence:

for $k \leftarrow 1$ **to** p **do**
    $\mathbf{r}_k = \mathbf{r} + \mathbf{X}_k\hat{\boldsymbol{\beta}}_k$;
    if $\|\mathbf{X}_k^t\mathbf{r}_k\|_F < \lambda\gamma_k$ **then**
        $\hat{\boldsymbol{\beta}}_k \longleftarrow 0$;
    end
    else
        $\hat{\boldsymbol{\beta}}_k \longleftarrow \left[\mathbf{X}_k^t\mathbf{X}_k + (\frac{\lambda\gamma_k}{\theta} + 2\alpha)\mathbf{I}\right]^{-1} \mathbf{X}_k^t\mathbf{r}_k$;
        where θ is the root of $\| \left[\mathbf{D}_k^2\theta + (\lambda\gamma_k + 2\alpha)\mathbf{I}\right]^{-1} \mathbf{D}_k\mathbf{U}_k^t\mathbf{r}_k\|_F = 1$
    end
    $\mathbf{r} \longleftarrow \mathbf{r}_k - \mathbf{X}_k\hat{\boldsymbol{\beta}}_k$;
end
return $\hat{\boldsymbol{\beta}}_1, \ldots, \hat{\boldsymbol{\beta}}_p$

practice, because we are fitting the group-lasso along a sequence of λ, we will initialize $\hat{\boldsymbol{\beta}}_1, \ldots, \hat{\boldsymbol{\beta}}_p$ with the estimates from the previous λ in the sequence. These "warm starts" give a significant speed advantage in our experience.

S2.2 Determining the group penalty modifiers γ_i

The γ_i in (7) allow us to have different penalties for different groups. This is useful because a larger group can be more likely to have a stronger correlation with the response than a small group, just by random chance. Having different penalties thus allows us to put different-sized groups on the same scale.

Recall that $\hat{\boldsymbol{\beta}}_k = 0$ if the following gradient condition is met:

$$\|\mathbf{X}_k^t \mathbf{r}_k\|_F < \lambda\gamma_k. \tag{S2-9}$$

It follows that we can determine an appropriate group penalty modifier by computing the expected value of the LHS if the signal were pure noise. Let $\boldsymbol{\epsilon} \sim (\mathbf{0}, \mathbf{I}_N)$, an N vector of white noise (we need only deal with the $T = 1$ case, since the $T > 1$ would be the same). Then we have

$$\begin{aligned}
\gamma_k^2 &= \mathbb{E}\|\mathbf{X}_k^t \boldsymbol{\epsilon}\|_F^2 && \text{(S2-10)}\\
&= \mathbb{E}\,\mathrm{tr}(\boldsymbol{\epsilon}^t \mathbf{X}_k \mathbf{X}_k^t \boldsymbol{\epsilon}) && \text{(S2-11)}\\
&= \mathbb{E}\,\mathrm{tr}(\mathbf{X}_k \mathbf{X}_k^t \boldsymbol{\epsilon}\boldsymbol{\epsilon}^t) \quad \text{(cyclic invariance of trace)} && \text{(S2-12)}\\
&= \mathrm{tr}(\mathbf{X}_k \mathbf{X}_k^t \mathbb{E}\boldsymbol{\epsilon}\boldsymbol{\epsilon}^t) \quad \text{(linearity of trace and expectation)} && \text{(S2-13)}\\
&= \mathrm{tr}(\mathbf{X}_k^t \mathbf{X}_k) && \text{(S2-14)}\\
&= \|\mathbf{X}_k\|_F^2. && \text{(S2-15)}
\end{aligned}$$

Therefore we take $\gamma_k = \|\mathbf{X}_k\|_F$, the Frobenius norm of $\mathbf{X}_k$. Note that if $\mathbf{X}_k$ is orthonormal, then $\gamma_k = \sqrt{p_k}$, which is the penalty modifier proposed in [2].

References

1. Tseng P (2001) Convergence of block coordinate descent method for nondifferentiable maximization. Journal of Optimization Theory and Applications 109: 474-494.

2. Yuan M, Lin Y (2006) Model selection and estimation in regression with grouped variables. Journal of the Royal Statistical Society - Series B: Statistical Methodology 68: 49-67.
